# How different types of environmentalists are perceived: changing perceptions by the feature

**DOI:** 10.3389/fpsyg.2023.1125617

**Published:** 2023-11-09

**Authors:** Karolin Kibele, Miriam Rosa, Milan Obaidi

**Affiliations:** ^1^Instituto Universitário de Lisboa (ISCTE-IUL), Lisbon, Portugal; ^2^Instituto Universitário de Lisboa (ISCTE-IUL), CIS-IUL, Lisbon, Portugal; ^3^University of Copenhagen (KU), Copenhagen, Denmark; ^4^University of Oslo (UiO), Oslo, Norway

**Keywords:** environmentalists, stereotypes, conjoint experiment, social identity, US residents

## Abstract

**Introduction:**

Previous research found stereotypes of environmentalists as barriers to public engagement and identification with environmentalism. Yet, there is limited understanding of the distinct attributes of an environmentalist that influence public perceptions and self-identification. In our research, we address this knowledge gap by analyzing reactions to a range of fictional environmentalist profiles.

**Methods:**

We investigated how multiple features of these profiles (e.g., gender, occupation, type of pro-environmentalism) influenced stereotypes (such as competence, friendliness, and trustworthiness), perceived typicality, and participants’ self-identification with the described profiles, using a novel conjoint experiment approach with 678 US residents.

**Results:**

We found that profiles described as women, Asians, working as a cleaner or office clerk, and politically moderate or liberal, exhibiting private to moderate environmental behaviors and global environmental concerns, were generally perceived as more typical for environmentalists. Moreover, participants most identified with profiles depicted as women, in a cleaner occupation, and exhibiting private pro-environmental behaviors. Atypical profile descriptions, based on prior research, enhanced participants’ impressions only when associated with private pro-environmental behaviors or the cleaner occupation.

**Discussion:**

We introduce new avenues in impression formation research and the use of conjoint analyses in psychological research; moreover, we contribute valuable input to the environmental movement regarding message framing considering the source and content relative to the targeted audience.

## 1. Introduction

Environmentalism has become one of the most polarizing and politicized issues in the United States today (Feygina et al., 2010; McCright and Dunlap, 2011; Pew Research Center, 2020). In 2021, only 41% of US citizens identified as “environmentalists,” a stark decline from 78% in 1991 (Gallup, 2021). Beyond the increasingly polarizing political debate on environmental issues in the United States, one explanation for the decreasing identification focuses on an increase in negative stereotyping (e.g., being aggressive, stubborn, or eccentric) against people who think of themselves as environmentalists or environmentally conscious (Stewart and Clark, 2011; Bashir et al., 2013; Klas et al., 2019). Further, US ethnic and racial minorities as well as economically disadvantaged groups are, contrary to their actual concern, perceived by the US society as least concerned about the environment and continue to be poorly represented in environmental organizations (Pearson and Schuldt, 2014; Taylor, 2014; Hiltner, 2019). This underrepresentation is particularly troubling since these marginalized and economically challenged communities bear a disproportionate burden of environmental risks (Mohai et al., 2009; Timmons Roberts et al., 2018). Termed as a “diversity crisis” (Pearson and Schuldt, 2014, p. 1034), this imbalance stems from enduring inequalities such as those in opportunities and education, coupled with unconscious biases in hiring practices and stereotypes, particularly portraying racial-ethnic minority groups as unconcerned (Taylor, 2014; Hiltner, 2019).

Based on previous research, an environmentalist can be defined as someone dedicated to protecting and enhancing the environment through different avenues, such as conservation or preservation (Tesch and Kempton, 2004; Bashir, 2010; Klas, 2016). In this study, we assumed that preexisting negative perceptions and stereotypes toward environmentalists prevent the general public from identifying, engaging, or supporting them (Bashir et al., 2013; Pearson et al., 2018). By mapping the underlying perceptions and impressions that US residents have of environmentalists (e.g., concerning competence, friendliness, and trustworthiness), we aimed to understand how people relate, both positively and negatively, to specific attributes associated with environmentalists. Moreover, we explored to what extent people’s personal characteristics (e.g., political orientation, social class) affect their perceptions of environmentalists. Gaining more knowledge on these patterns contributes to the literature on environmentalist stereotyping and enhances our understanding of strategies to boost diversity among members and garner broader public support for environmental movements.

Previous research connected climate change and environmental justice^1^ research with socio-psychological approaches through the study of intergroup processes in the United States (Pearson and Schuldt, 2018; Swim and Bloodhart, 2018). For example (negative), stereotypes toward environmentalists were identified as barriers to social change (Bashir et al., 2013), and people preferred pro-environmental messages coming from members of the same political party (Bolsen et al., 2019). Based on this research, Stenhouse and Heinrich (2019) applied a conjoint analysis to examine individuals’ inclinations toward various personal attributes of climate activists and to determine how responses varied based on political party affiliation. However, their study did not delve into the stereotypic associations related to environmentalists, the factors driving identification with them, or the influence of perceivers’ own characteristics. In the present study, we applied a conjoint analysis through a multidimensional rating experiment. Our goal was to investigate patterns of public impressions, perceptions of the prototypical environmentalist, and individuals’ identification with environmentalists. Crucially, we sought to understand the interplay between multiple identity dimensions of environmentalists on participants. These dimensions included social class, race/ethnicity, and political orientation.

More precisely, the study aims at understanding which attribute values of fictitious environmentalists (a) inform stereotypical dimensions (e.g., competence, warmth, and morality), (b) are considered more typical of environmentalists, (c) and elicit more self-identification of participants with environmentalists. It also aims at expanding previous research on environmentalists that deviate from stereotypical depictions (i.e., that are atypical according to stereotype-literature), testing whether the presentation of environmentalists with attributes inconsistent with these stereotypes (as opposed to consistent attributes) enhances positive impressions and identification with them.

## 2. Literature review

In the following, a comprehensive theoretical foundation for our study will be provided, offering insights into how the dimensions of stereotypes, group identities, self-categorizations, and broader cultural norms interplay to shape public engagement with the environmental movement.

Stereotypes represent generalized beliefs about specific groups (Stangor and Lange, 1994). There is some consensus in the literature suggesting that these beliefs are primarily shaped along two dimensions. Specifically, research on the *Stereotype Content Model* (SCM; Fiske et al., 2002) has shown that positive and negative evaluations of other people and groups can be assessed through the two dimensions *warmth* (i.e., being warm, sociable, friendly) and *competence* (i.e., being competent, agentic, intelligent). Different dimension combinations result in distinct intergroup emotions (e.g., pity, envy, admiration, and contempt) and, consequently, in different forms of prejudices. By examining the content of people’s perceptions of different groups (highlighted by characteristics like age, gender, occupation, ethnicity, race), Fiske et al. (2002) mapped prevalent societal patterns and tendencies in stereotyping. For example, they showed that men were primarily perceived as competent but not warm leading to feelings of admiration and envy. In contrast, women were seen as both competent and warm resulting in admiration. In terms of race and ethnicity, Fiske et al. found distinct stereotyping patterns in the United States. For example, racial-ethnic minority groups like Hispanics, Native Americans, and Black individuals were typically perceived as moderately warm and competent. Asians were stereotypically seen as competent but lacking in warmth, while Whites were generally perceived as both highly warm and competent. Stereotypically, higher status is often associated with competence, while competition tends to correlate with a low level of warmth perceptions. This provides the basis for understanding how certain groups might be liked or disliked, and respected or disrespected.

The SCM could help explain why certain environmentalists are perceived as more typical or more likable. For instance, Eckes (2002) found that women were frequently stereotyped as warm but not as competent, whereas men were perceived as competent but not warm. Also, when considering political stereotypes, individuals who identify as politically liberal are often perceived as warm but not competent. Hence, if environmentalists are described as women or politically liberal, they might be perceived as warm (friendly, nurturing) but not necessarily as competent. When considering racial stereotypes, Lin et al. (2005) found that Asians are often stereotyped as competent but not as warm in American society. Conversely, environmentalists described as Asians might be viewed as more competent but lacking in warmth. These perceptions may significantly influence the audience’s inclination to identify with and support the environmental movement. Occupational stereotypes, such as those from Durante et al. (2013) which explored 37 countries, further highlight how certain professions, when viewed as social groups, are often stereotyped in terms of warmth and competence, which in turn affects the perceptions of environmentalists affiliated with these professions.

Nevertheless, attributions of warmth and/or competence are shaped not only by individuals’ sociodemographic characteristics, but also by the extent of their engagement in pro-environmental activities with which they are associated (Bashir, 2010; Bashir et al., 2013; Castro et al., 2017). Using the SCM framework by Fiske et al., 2002, Castro et al. (2017) found that fictitious individuals expressing strong or radical environmentalism were stereotyped as less warm, though they were still regarded as equally competent. In comparison, those who engaged in private environmental actions (e.g., organic purchase, recycling, water, and energy saving) were positively evaluated on both dimensions. In a related study examining the discourse of environmentalists, participants preferred a concessional yes-but approach (Castro et al., 2017). This preference underscores a tendency toward more moderate and conciliatory pro-environmental stances. However, it is worth noting that the authors did not incorporate identity markers, like gender when characterizing the fictitious individuals.

Moreover, there is an increasing interest in a third dimension of stereotype content. In particular, the stereotypes traditionally categorized under warmth not only encompass friendliness and sociability, but also trustworthiness, which includes aspects like honesty, sincerity, and morality. Notably, these aspects have been demonstrated to be orthogonal (Leach et al., 2007; Ellemers et al., 2013; Landy et al., 2016). Such studies indicate that environmentalists are deemed more trustworthy or moral when they adopt a radical discourse compared to a moderate one. Their perceived warmth hinges on the societal consensus surrounding the discourse topic, while their perceived competence remains constant (Castro and Rosa, 2023). Intriguingly, younger environmentalists are not perceived as lacking in competence or warmth compared to their older counterparts. However, they are considered less trustworthy (Farinha and Rosa, 2022). Therefore, the present study extends this previous research by examining the influence of multiple personal attributes of environmentalists (e.g., gender identity, race/ethnicity, political orientation) on participants’ perception and their identification with them.

Together, these studies provide ample support for the application of the SCM to understand how different characteristics of environmentalists might influence their perception in terms of warmth and competence and, subsequently, the willingness of individuals to engage and identify with the environmental movement.

In terms of identification processes, stereotypes can play an important role. Stereotypes are mental constructs that often deviate from the true characteristics of the ideal or average group member. Instead of providing a comprehensive or accurate portrayal, they can sometimes offer a distorted, overly simplistic, or even negative view of a social category (Hilton and von Hippel, 1996). Previous research on stereotypes associated with environmentalists has revealed a range of positive and negative perceptions held by the general public. Research has shown that positive attitudes toward the prototypical environmentalist, as well as identifying as an environmentalist, are linked to pro-environmental behaviors and policy preferences (Ratliff et al., 2017; Brick and Lai, 2018). Given this, stereotypes might help explain the social barriers some societal groups face when considering identification with or engagement in the environmental movement (Bashir et al., 2013; Swim and Bloodhart, 2018; Klas et al., 2019). The Social Identity Theory (SIT) posits that individuals derive a portion of their self-concept from the social groups they belong to, leading to a bias toward in-group members and discrimination against out-group members (Tajfel and Turner, 1979). This theory is particularly relevant to our study as it highlights how people’s identification with the profiles of environmentalists might be influenced by their own group memberships. For instance, if participants see themselves as part of a “liberal” group, they might be more inclined to identify with environmentalists that are also politically liberal. SIT can therefore offer insights into how stereotypes and social identities interplay in shaping public engagement with environmentalism. For example, individuals avoid affiliating (which is a form of identification) with environmentalists when they perceive them as militant/aggressive or eccentric/unconventional (Bashir et al., 2013). This is in line with the findings of Klas et al. (2019) illustrating that behaviors at an individual/private level (e.g., recycling) can be perceived more positively (e.g., as valuing nature), whereas collective actions or other public sphere behaviors (e.g., demonstrations) are judged negatively (e.g., as aggressive and stubborn). SIT involves processes of categorization (how we see ourselves as members of a given group), identification (the emotional significance of that membership) and social comparison [how well or worse-off is the own group (ingroup) compared to other (out-groups)]. The Self Categorization Theory (SCT; Turner et al., 1987) deals precisely with categorization processes and brings forward how important typicality is: people have a representation (prototype) of what a typical group member is for any given social group, and group members can differ in the extent to which they are typical of the group.

Perceptions of environmentalists may vary depending on different attributes and which traits are typically associated with members of this social category. Considering that these perceptions are mostly negative (and, thus, social comparison processes are not in their favor), they represent possible reasons why people refuse to identify with environmentalists or to participate in pro-environmental behaviors, as well as why environmentalists might hold back on public engagement and advocacy. In the US context, environmentalists are among the most politicized groups and they are typically associated with the Democratic party and left-wing ideology (Merkley and Stecula, 2018). Studies showed that individuals who are concerned about the environment or engage in environmentally conscious behaviors are typically perceived as more feminine (Brough et al., 2016; Swim and Bloodhart, 2018). Pearson et al. (2018) found that the typical perception of environmentalists among diverse societal groups in the United States included the features of White and highly educated. However, when contrasting these perceptions to the reported self-identification of people from different racial-ethnic groups, results revealed that minority groups (e.g., Latinos/as and Asian Americans) identified themselves more as environmentalists than Whites. Pearson et al. describe this phenomenon as an *environmental belief paradox*, meaning the tendency to (self-) stereotype, misperceive, and underestimate low-income and underrepresented groups’ identification with environmentalists, when those groups are most concerned and vulnerable to negative environmental impacts. The environmental belief paradox can be reduced by exposing diverse participants to images and descriptions of racially diverse (vs. non-diverse) members of environmental organizations (Pearson et al., 2018). Pearson et al. explained this effect through the presence of diversity cues as enhancing the perceptions of inclusion and belonging among the underrepresented study participants (Purdie-Vaughns et al., 2008).

Bashir et al. (2013) showed that typical environmentalists were associated with militancy and eccentricity, resulting in a reduced receptiveness toward activists and the social and behavioral changes for which they advocated. Interestingly, these results were less pronounced for portrayals of environmentalists depicted with descriptions that challenge conventional activist group stereotypes (e.g., being pleasant and approachable). Such portrayals elicited more positive responses and a heightened willingness to associate with them. Thus, it is not just the group membership that influences participants’ impressions, it also influences the degree to which environmentalists align with or deviate from the currently prevailing group stereotypes. Building on this research, our study integrates various profile attributes deemed atypical in existing literature. We aim to examine whether perceptions of these environmentalists are more favorable compared to typical profiles. For instance, we introduced the non-binary gender identity as one of the descriptive attributes of environmentalists.

Lastly, our study suggests a strong influence of cultural stereotypes and social norms on people’s impressions and self-identification with environmentalists. This framework considers that the broader cultural context, including societal stereotypes about certain professions, genders, or ethnicities, play a significant role in shaping perceptions. In our study, the inclusion of stereotypes related to women, Asians, or specific occupations indicative of social class (e.g., working as cleaners or office clerks) could contribute making these profiles appear as more typical or relatable environmentalists. Similarly, the norms associated with political ideologies might affect how typical or likable an environmentalist is perceived, affecting people’s willingness to engage with environmentalism. If participants show tendencies to self-identify most with environmentalists at particular attribute levels, this may be understood as *self-defining* and *self-investing* components of identification (Leach et al., 2008). In this respect, participants may perceive themselves (i.e., individual self-stereotyping) and their ingroup (i.e., in-group homogeneity) as similar to the environmentalists described in a certain way. According to Leach et al. (2008), participants may: (1) feel positively toward these environmentalists (i.e., *satisfaction*); (2) feel a sense of belonging and attachment to certain profiles (i.e., *solidarity*); and (3) perceive them as central to their self-concept, thus, being more aware of ingroup threats (i.e., *centrality*).

Pioneering a new line of research, Stenhouse and Heinrich (2019) presented numerous profile variations and simultaneously tested many attribute factors through the application of a conjoint experiment. They discovered that the most significant effects were linked to the activists’ viewpoints on climate change, the frequency with which they pressured others to act on climate change, their stance on gun control, and their party affiliation. They concluded that to enhance the general public’s appeal toward climate activists, they should be depicted as friendly and non-militant. While Stenhouse and Heinrich (2019) expanded our understanding of public perceptions of environmentalists, they did not consider other identity-relevant dimensions in their research.

Thus, people’s impressions and stereotypes of environmentalists can be influenced by various factors including their labels, attribute traits, or actions (Bashir et al., 2013; Castro et al., 2017; Klas et al., 2019). The present study contributes to the social identity and stereotype literature by examining public impressions and evaluations of environmentalists along the stereotype dimensions of competence, warmth, and morality. Moreover, we aim to assess participants’ self-identification with environmentalists and their perceptions of environmentalists’ typicality based on various attributes.

By incorporating and analyzing multiple relevant identity dimensions of environmentalists (e.g., gender, social class, race/ethnicity, political orientation), this study takes an intersectional approach (American Psychological Association, 2017). To conduct a one-by-one examination of multiple factors, we apply an experimental approach (with systematic variation and randomization)—that is, *conjoint analysis* (Hainmueller et al., 2014). Conjoint analyses allow the merger of directional expectations for some attributes and an exploratory approach for others. As we aim to examine a large number of identity factors as well as the often-ambiguous nature of impressions and stereotypes, we derived some directional hypotheses to address our research aims.

Drawing from existing literature on attributes associated with environmentalists, we anticipate that while female and White environmentalists will be perceived as both warm and competent, those who are Asian, male, or associated with high-status occupations will be seen as competent but not necessarily warm (H1.1a). Concerning the actions of environmentalists, we anticipate that those described as more radical will be perceived as less warm albeit equally competent. In contrast, environmentalists characterized by private pro-environmental behaviors will likely be viewed warmer while maintaining a similar level of perceived competence (H1.1b). Generally, environmentalists that could be perceived as eccentric or confrontational will likely be viewed negatively (H1.1c). Furthermore, we expect that White female environmentalists of middle social status with liberal political orientation, especially those who are more radically active, will be most frequently perceived as typical environmentalists (H1.2). Moreover, we expect that participants will most likely identify themselves with environmentalists that show private pro-environmental behaviors (H1.3). In terms of positive impressions and identification with environmentalists based on their typicality, and similar to Bashir et al. (2013) results, we expect to find positive effects across all dependent variables through the inclusion of stereotype-inconsistent attributes (H2).

**Figure 1:**
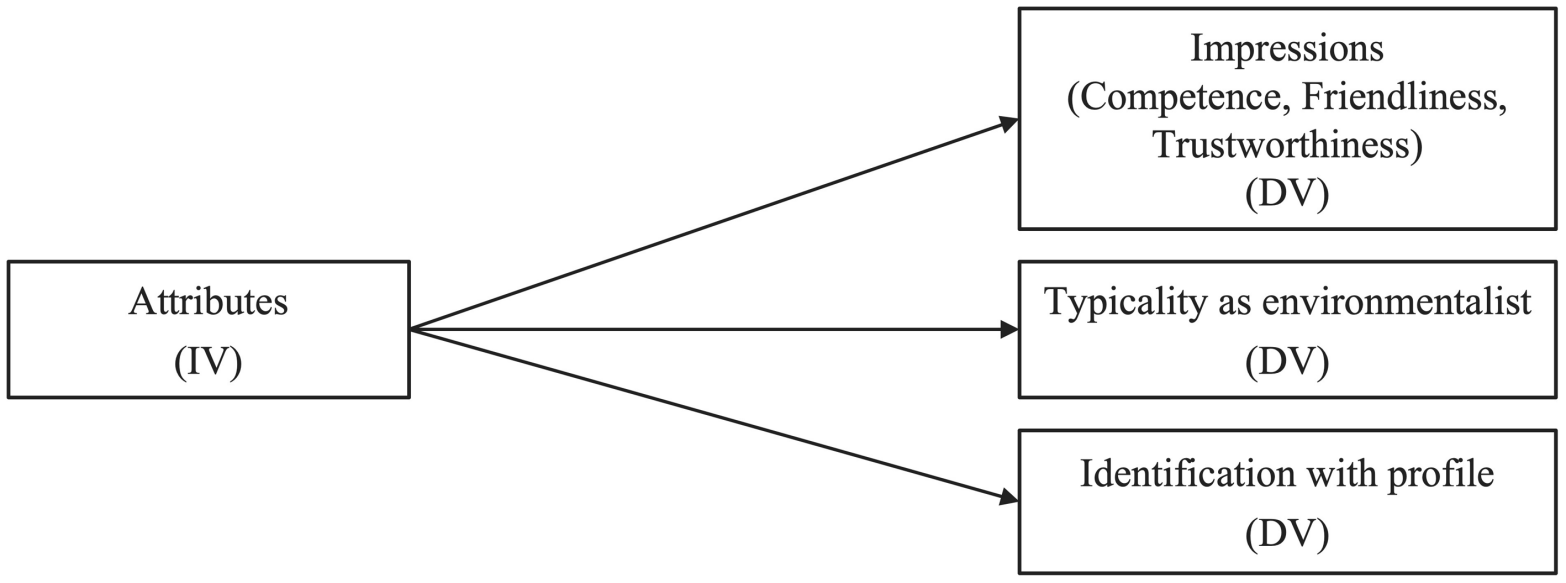
Simple study model indicating the influence of environmentalists’ profile attributes on participants’ impressions, perceived typicality as environmentalist, and self-identification.

The presented hypotheses are examined with a sample of US residents; therefore, the present study provides new insights into the perceptions of environmentalists in the United States. Figure 1 shows the profile attribute values representing the independent variables (IV); the participants’ evaluations were assigned as dependent variables (DV).

## 3. Materials and methods

### 3.1. Conjoint analysis

Conjoint experimental designs, originally developed by Luce and Tukey (1964), have been traditionally used in marketing research but recently introduced to the field of political science as well (e.g., Doherty et al., 2019; Knudsen and Johannesson, 2019; Carey et al., 2020). Applied to psychological research, this approach allows the investigation of people’s responses to a multitude of complex and interacting influences. Like vignettes, conjoint designs describe a product or person, subsequently referred to as *profile*, based on the different characteristics presented to respondents in a table format (Stenhouse and Heinrich, 2019). *Attributes* refer to the name of features or characteristics that describe the profiles, consisting of *levels* or *values* representing the different choices for each attribute (Qualtrics XM support, 2021). The two profiles are generated completely at random assigning “a value for each attribute, and the order of attributes randomized as well” (Stenhouse and Heinrich, 2019, p. 344). In a conventional experimental approach in psychology, different experimental conditions are presented to participants or separate groups. However, in conjoint experiments, fully randomized attribute orders and values are presented to each individual participant (Stenhouse and Heinrich, 2019). Hence, this method does not require experimental participant subgroups or separate conditions.

Being a multidimensional choice or rating experiment, this method allows a fully randomized factorial and between-subjects design that simultaneously tests the influence of various factors on participants’ evaluations of environmentalists’ profile descriptions. These evaluations are used to calculate the participants’ impressions and tendencies within individual profile attributes as well as group differences between the participants (Leeper et al., 2020). Furthermore, the use of conjoint analysis allows for a detailed examination of individual effects, accommodating both directional hypotheses and exploratory questions, making it well-suited for our research objectives. In summary, conjoint analysis has shown to be a functional, practical, and efficient method that has not yet received adequate attention in psychological research. For more information on the statistical analysis of conjoint designs, the assumptions of conjoint analysis, and the method’s strengths and benefits, please see the Supplementary materials.

### 3.2. Participants

For the present study, we recruited 1,452 US residents. Our target sample size followed the recommendations and model-based statistical power calculations for conjoint designs by Stefanelli and Lukac (2020), requiring a minimum of *N* = 620 participants to ensure adequate statistical power (1 – ß = 0.80). We recruited our sample via convenience sampling (e.g., in social media groups, private social, and academic networks) and the Amazon Mechanical Turk (MTurk) crowdsourcing platform, adhering to recommended practices (Black, 2021).^2^ Participants either received the option of qualifying to win a $50 gift certificate (convenience sample) or compensation of $2 for their completed participation (MTurk sample). To prevent respondents from being unconscientious during the survey, participation qualifications and control questions were integrated into the questionnaire.

Participant responses were collected between April 13 and May 20, 2021. Out of the original 1,452 responses, 774 were removed due to incomplete survey responses, failed attention checks, and rapid completion (under 5 mins), leaving *N* = 678 valid responses for statistical analysis. From those, *n* = 364 (53.7%) were recruited through convenience sampling and *n* = 314 (46.3%) through Amazon MTurk. Separate analyses were conducted for each sample as detailed in the results section and subsequently discussed. Participants were between 18 and 85 years of age (*M* = 34.26, *SD* = 12.16). The gender distribution was as follows: of the total sample, 317 participants (46.8%) identified as female, 352 (51.9%) as male, three (0.4%) as agender or non-binary, one (0.1%) chose “prefer not to say,” and five (0.7%) did not provide a response. Participants’ religiosity was assessed using a scale from 1 (Not religious at all) to 7 (Very religious), yielding a mean score of 3.89 (*SD* = 2.20). Their political orientation was gauged on a scale ranging from 1 (Strongly liberal) to 7 (Strongly conservative), with an average score of 3.65 (*SD* = 1.86). Comprehensive socio-demographic details are provided in Table 1.

**Table 1:** Socio-demographic data with sample sizes and percentages.

Sociodemographic category	Sample size and percentage of participants	
	*n*	*%*
Race/ethnicity		
White/Caucasian	503	74.2
Black or African American	65	9.6
Hispanic or Latino	52	7.7
Asian or Asian American	32	4.7
Middle Eastern	1	0.1
American Indian or Alaska Native	9	1.3
Multi-ethnic/multiracial (accumulated)	15	2.2
Prefer not to say	1	0.1
Self-assessed social class		
Lower class	92	13.6
Middle class	525	77.4
Upper class	61	9.0
Yearly household income		
Less than $10,000	31	4.6
$10,000–$29,999	84	12.4
$30,000–$49,999	148	21.8
$50,000–$69,999	136	20.1
$70,000–$89,999	88	13.0
$90,000–$119,999	65	9.6
$120,000–$149,999	42	6.2
$150,000–$179,999	27	4.0
$180,000–$209,999	13	1.9
More than $210,000	30	4.4
Didn’t respond	14	2.1

### 3.3. Procedure

The study was carried out in accordance with the declaration of Helsinki regarding research with human beings. The local ethics committee approved the research (information omitted for blind review) and pre-registered on AsPredicted.org.

The applied conjoint analysis was constructed and administrated as a 25-min online questionnaire via the Qualtrics survey platform (Qualtrics, 2005). After responding to an informed consent, the participants were presented with separate conjoint modules describing a total of eight environmentalist profiles in a table format. Throughout the study, we provided participants with our operational definition of environmentalists as described previously. As is standard in conjoint designs, these descriptions were fully randomized. Subsequently, participants evaluated them based on their impressions, perceived typicality as environmentalists, and the degree of self-identification participants felt with them. Aside from the conjoint variables, participants’ socio-demographic data and their attitudes regarding environmentalism were recorded. Finally, participants were fully debriefed and given the option to leave comments.

### 3.4. Materials

**Figure 2:**
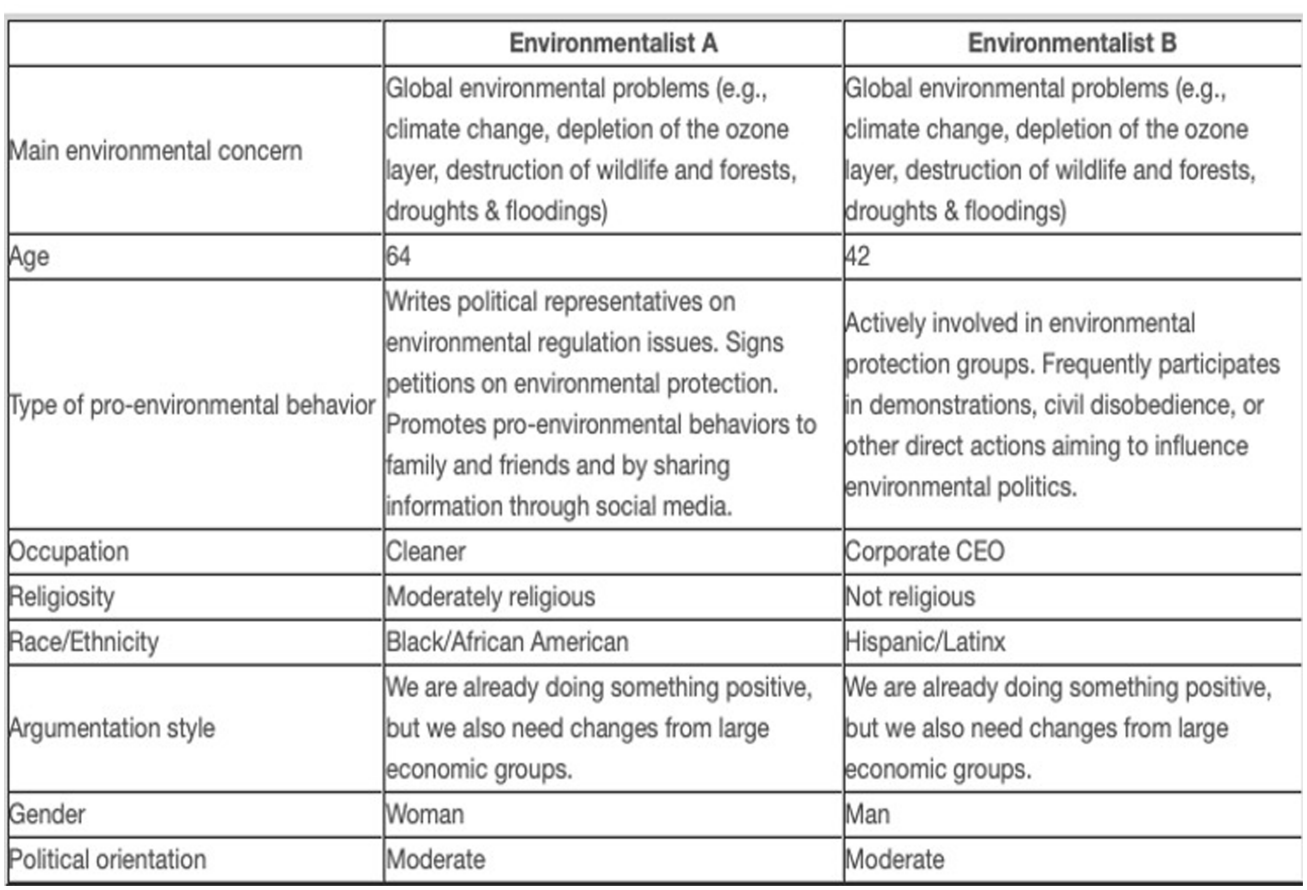
Example conjoint table describing two environmentalists **(A,B)**.

The acquired survey data encompassed the independent, dependent, and subgroup variables within the examined model. The provided experiment incorporated four conjoint modules. Each module featured a conjoint table that described two fictitious environmentalist profiles (A and B see Figure 2). The profiles were characterized by nine attributes each falling within specified categories. These attributes were randomly ordered with values chosen from a pool of potential attribute levels as independent variables (IVs). The conjoint table was followed by five rating tasks to capture participants’ impressions (on the dimensions of competence, warmth, and morality), typicality of environmentalists, and self-identification with the profiles as dependent variables (DVs). Furthermore, socio-demographic data, as well as environmental standpoint and optional identity variables were assessed for further possible analyses. The online survey was developed using HTML and JavaScript coding to create the conjoint experiment.^3^

#### 3.4.1. Stimuli

The profile attributes and attribute values^4^ were selected based on previous research related to stereotypes of people who engage in pro-environmental behaviors or are labeled as environmentalists. To approximate a realistic portrayal of an environmentalist, the profile descriptions incorporated attribute categories such as “Age,” “Gender identity,” “Race/Ethnicity,” “Occupation,” “Religiosity,” “Political orientation,” “Type of pro-environmental behavior,” “Main environmental concern,” and “Argumentation style.” Due to design restrictions imposed by statistical power calculations (Stefanelli and Lukac, 2020), the maximum number of values per attribute was limited to four. The chosen values were designed to encompass both stereotype-consistent and stereotype-inconsistent descriptions. The full text of the profile attributes and their values are provided below in Table 2. On another note, for a traditional experimental setup, the factorial structure for the independent variables would consist of a 3 × 3 × 3 × 4 × 3 × 3 × 3 × 2 × 2 (multiplied attribute values) design including a total of 11,664 experimental conditions. In turn, the application of a conjoint experiment allowed the testing of all these factors within one experimental condition with a substantially reduced sample size.

**Table 2:** Full text of all profile attributes (variables) and attribute values (levels).

Attribute	Value
Age	23
	42
	64
Gender identity	Woman
	Man
	Non-binary
Race/ethnicity	White
	Black/African American
	Hispanic/Latinx
	Asian
Occupation	Office clerk
	Corporate CEO
	Cleaner
Religiosity	Not religious
	Moderately religious
	Very religious
Political orientation	Liberal
	Moderate
	Conservative
Type of pro-environmental behavior	Actively involved in environmental protection groups. Frequently participates in demonstrations, civil disobedience, or other direct actions aiming to influence environmental politics.
	Writes political representatives on environmental regulation issues and signs petitions on environmental protection. Promotes pro-environmental behaviors and shares information with family, friends, and through social media.
	Prefers purchasing environmentally friendly goods, such as local organic food, or recycled products. Separates garbage at home and uses (natural) resources responsibly, like avoids wasting food, energy, or water, or drives less by car.
Main environmental concern	Global environmental problems (e.g., climate change, depletion of the ozone layer, destruction of wildlife and forests, droughts & floodings)
	Neighborhood environmental problems (e.g., too much trash & noise, lack of access to natural areas or grocery stores, proximity to polluting industrial sites)
Argumentation style	What we are doing is not enough. We need fundamental changes from large economic groups.
	We are already doing something positive, but we also need changes from large economic groups.

*Age and Gender Identity.* Three age (e.g., 23) and gender identity (e.g., woman and non-binary) values were included representing different social groups in US society. To the best of our knowledge, the “non-binary” gender identity has not been previously explored in the literature on environmentalist stereotypes.

*Race/Ethnicity and Occupation.* As reviewed previously, traits related to race, ethnicity, and socio-economic status are relevant dimensions associated with existing public perceptions of environmentalists (Pearson et al., 2018). Unfortunately, due to sample size considerations and power, only the four largest racial and ethnic groups in the United States could be included (US Census Bureau, 2019). The selection of occupation aimed to signify socio-economic status. This approach was adopted to avoid random combinations of multiple socio-economic variables, which might have produced implausible profile descriptions (for instance, a doctor possessing only a high school degree). Such inconsistencies could potentially lead to participant confusion, as highlighted by Hainmueller et al. (2014).

*Religiosity and Political Orientation.* Religion or religiosity, while a significant factor in shaping social identity in the United States (Arbuckle, 2017), has yet to be extensively explored in the context of environmentalist stereotype literature. Again, to avoid unusual attribute combinations and to keep the limit of four levels for each attribute, we chose to include three levels of religiosity (not, moderately, and very religious) instead of religious affiliation. Moreover, political orientation representing one of the most significant and polarizing factors influencing US residents’ views on environmentalism, was included with three distinct values (liberal, moderate, and conservative; Merkley and Stecula, 2018).

*Type of Pro-Environmental Behavior and Main Environmental Concern.* People’s understanding and impressions of environmentalists are influenced by the nature of the pro-environmental behaviors they exhibit. Therefore, the inclusion of three distinct behavioral descriptions (indicating radical, moderate, and private behaviors) for environmentalists was intended to provoke varied responses. Furthermore, diverse people have different environmental concerns and therefore might align more with global or local concerns (Mohai and Bryant, 1998). Both forms of environmental concern were included as attribute values.

*Argumentation Style.* Environmentalists’ discourse can have radical and moderate argumentative styles (see Uzelgun et al., 2015; Castro et al., 2017; Castro and Rosa, 2023). We included environmentalists’ argumentation style via two distinct messages. One used a moderate and concessional “yes-but” discourse, suggesting that while significant efforts are underway, they remain insufficient. In contrast, the second employed a more confrontational and non-compromising “no-no” discourse, emphasizing the need for immediate and radical action.

#### 3.4.2. Measures

After being presented with the conjoint tables containing the above-explained stimuli, participants were asked to rate their impressions of the described environmentalists on a 7-point Likert scale (“1 = Strongly disagree” to “7 = Strongly agree”). As is standard for conjoint experiments (Hair, 2014), the participants’ impressions were measured using single-item constructs. The specific measures used in the questionnaire are available in the Supplementary materials.

*Stereotypical Impressions.* Using the dimensions established by Fiske et al. (2002) and Leach et al. (2007), participants’ impressions were assessed in terms of competence, warmth, and morality. Participants were asked to indicate their level of agreement or disagreement regarding whether the presented profiles were “friendly” (representing warmth), “competent” (representing competence), and “trustworthy” (representing morality). In the following, the measure “friendliness” refers to the dimension warmth and the measure “trustworthiness” to the dimension morality.

*(Proto)Typicality and Self-Identification.* Participants were asked to indicate their level of agreement or disagreement concerning the typicality of the presented profile as an environmentalist, as well as their personal identification with them.

*Environmentalism, Socio-demographic Data, and Other Identity Variables.* Participants provided information regarding their own environmental beliefs, socio-demographic details, and affiliations with other groups. Their perspective on environmentalism was assessed through how strongly they identified as an environmentalist, their personal level of concern about environmental issues, their own pro-environmental actions, and which environmental problem they deemed most important. The assessed socio-demographic questions concerned the participants’ self-assessed social class, race/ethnicity, age, gender identification, religious affiliation, religiosity, education, yearly household income, and political orientation.

### 3.5. Statistical analyses

For the statistical analyses, the dataset was prepared in SPSS, version 27.0 (IBM Corp., 2020). All hypotheses were then tested via conjoint analysis in R using the Cregg package (Leeper, 2020). Following the recommendation of Hainmueller et al. (2014), we conducted diagnostic checks to confirm that the assumptions for conjoint analyses were met (see Supplementary materials). Furthermore, external validity was ensured beforehand by assessing whether random attribute combinations could produce implausible profile descriptions. The generated randomization code was also put in place to avoid any unintended effects from the order of attributes.

First, we computed the Marginal Means (MMs) for each attribute value. These MMs represent the average ratings from all participants for each value, marginalized over all profile attributes. This approach offers insights into the estimates and patterns of participants’ impressions as recommended by Leeper (2020). Additionally, we formally tested for differences within one attribute category (between the attribute values’ MMs) by running omnibus *F*-tests using nested model comparisons. Unfortunately, the Cregg package (Leeper, 2020) did not provide the option of multiple comparisons for more than two levels/values; therefore, we could not simultaneously estimate the differences between all attribute values.

Then, we computed the average Marginal Component Effects (AMCEs). These indicate the effect sizes of each attribute value both within and in relation to its own attribute category. The AMCE values are calculated through the differences between all marginal means of one attribute category averaged by the marginal mean of the reference value. We applied values that were consistent with prior stereotype-literature (e.g., young, female, radical) as reference values for the AMCE calculations. Hence, the AMCEs provided patterns of participants’ impressions relative and conditional to the selected reference values as a baseline for each conjoint attribute. Moreover, their confidence intervals (CIs) indicated if there was a significant difference between stereotype-inconsistent and -consistent values.

## 4. Results

The results of the effects on profile attributes are presented visually by plots as well as through omnibus F-tests, with only significant results being reported. The exact numerical estimates, standard errors, and z-scores, as well as an overview of the descriptive statistics and correlations, can be found in the Supplementary materials.

Testing for differences between the two sample groups (MTurk sample and convenience sample), pairwise comparisons showed differences in the dependent variables: competence *F*(18, 5,388) = 1.71, *p* = 0.03, friendliness F(18, 5,388) = 3.77, *p* < 0.001, trustworthiness F(18, 5,388) = 3.74, *p* < 0.001 and typicality F(18, 5,388) = 1.87, *p* = 0.01. This limitation is discussed below.

**Figure 3:**
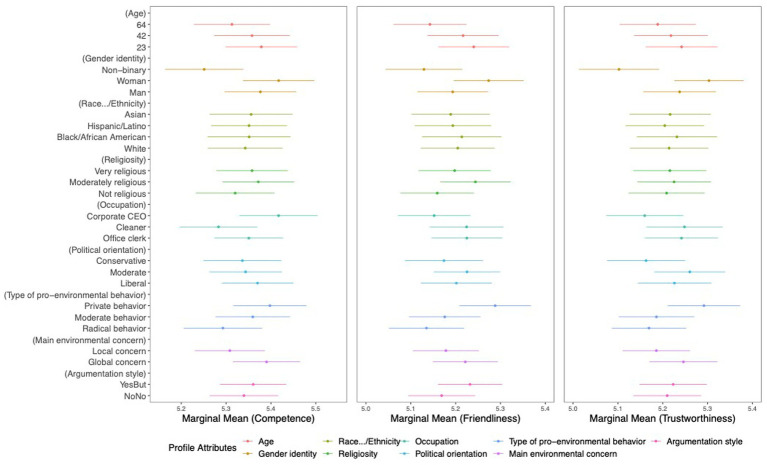
Marginal mean estimates for each attribute value on the profile ratings for competence, friendliness, and trustworthiness. The x-axis units are the original scale points for each dependent variable.

**Figure 4:**
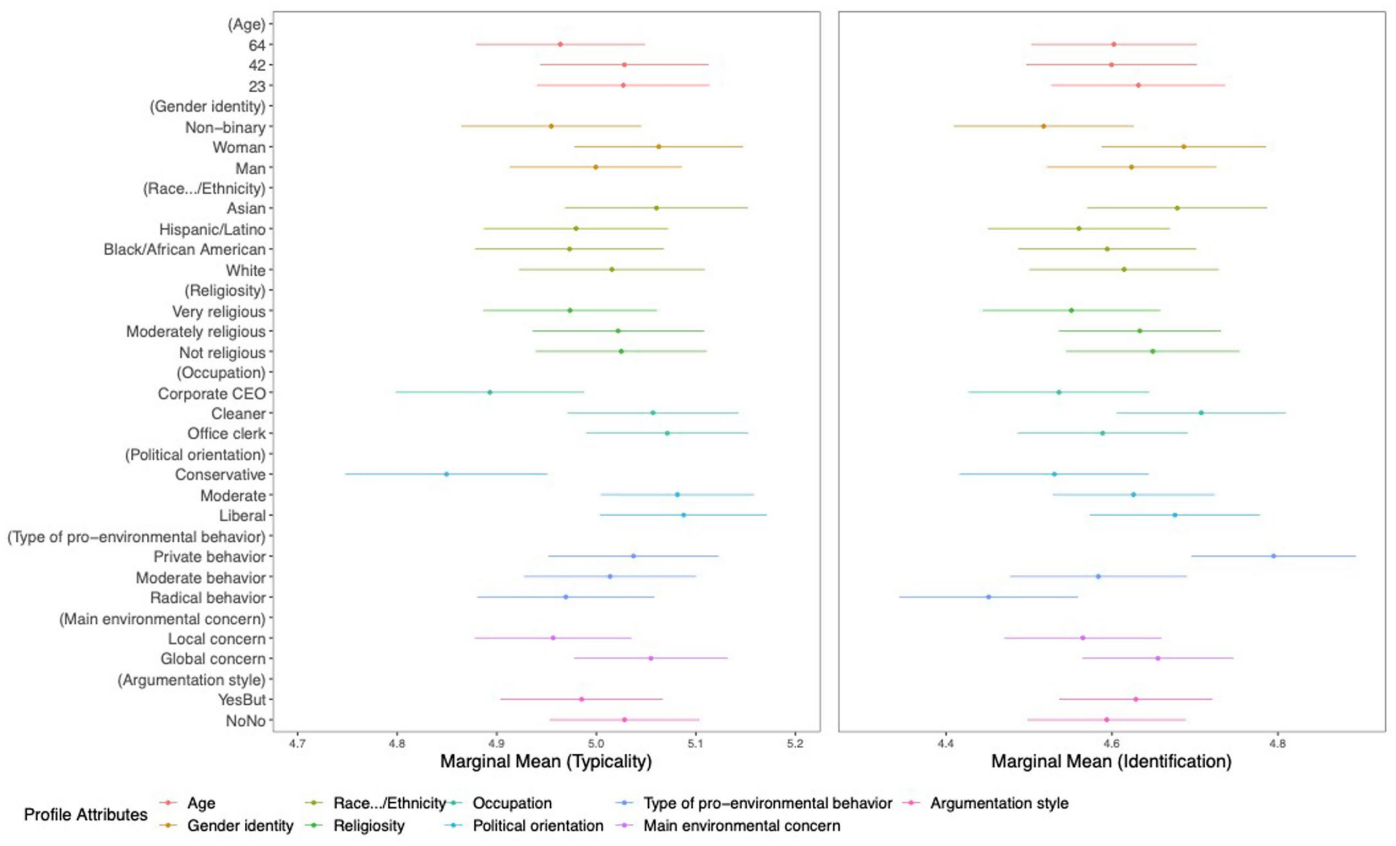
Marginal mean estimates for each attribute value on the profile ratings for typicality as environmentalist and self-identification with the profiles. The x-axis units are the original scale points for each dependent variable.

Figure 3 displays the results of the marginal means of the profile ratings for competence, friendliness, and trustworthiness, and Figure 4 displays the results of the ratings on profiles’ typicality as environmentalists and participants’ self-identification with the profiles. In each graphic, dots represent the marginal means, which are the estimates for every attribute value averaged across all participants. The horizontal lines on either side of the dots are the upper and lower limits of the mean dispersion. The x-axis units are the original scale points for each dependent variable.

Tendencies are visible in the ratings of the profiles’ competence, friendliness, and trustworthiness, and they vary in relation to the given profile attribute values within the given range (MMmin = 5.0, MMmax = 5.5). As shown in Table 3, significant mean differences between the values were found for some environmentalists’ attribute categories, suggesting that the environmentalists’ profiles were perceived more positively or more negatively when described with the listed profile attributes.

**Table 3:** Significant mean differences between the values by environmentalists’ attribute categories.

Variables	Competence	Friendliness	Trustworthiness
Age	–	*F*(4, 5,418) = 3.22, *p* = 0.012	–
Gender identity	*F*(4, 5,418) = 5.68, *p* < 0.001	*F*(4, 5,418) = 5.86, *p* < 0.001	*F*(4, 5,418) = 7.78, *p* < 0.001
Occupation	*F*(4, 5,418) = 3.04, *p* = 0.012	–	–
Political orientation	–	–	*F*(4, 5,418) = 3.09, *p* = 0.015
Type of pro-environmental behaviors	–	*F*(4, 5,418) = 4.87, *p* < 0.001	*F*(4, 5,418) = 3.63, *p* = 0.006

*Most positive environmentalist profile.* Environmentalists described as younger (most friendly), as a woman (most competent, friendly, and trustworthy), as a corporate CEO (only most competent), as politically moderate (most trustworthy), and as privately pro-environmental (most friendly and trustworthy).

*Most negative environmentalist profile.* Environmentalists described as older (least friendly), as non-binary (least competent, friendly, and trustworthy), working as a cleaner (least competent), as politically conservative (least trustworthy), and as radically pro-environmental (least friendly and trustworthy).

As seen in Figure 4, tendencies are visible in the ratings of environmentalists’ typicality (MMmin = 4.7, MMmax = 5.2) and participants’ self-identification with the profiles (MMmin = 4.5, MMmax = 4.9), which vary in relation to the profile attribute values in the given range. As shown in Table 4, significant mean differences between the values were found within the following environmentalists’ attribute categories on the outcome typicality as environmentalist and participants’ self-identification with the environmentalists’ profiles. This suggests that our sample perceived the presented profiles as most typical for environmentalists or identified most or least with them when described with the listed profile attributes.

**Table 4:** Significant mean differences between the values by environmentalists’ attribute categories.

Variables	Typicality	Self-identification
Age	–	–
Gender identity	*F*(4, 5,418) = 2.93, *p = 0*.020	*F*(4, 5,418) = 3.13, *p = 0*.014
Race/ethnicity	*F*(6, 5,414) = 2.37, *p = 0*.027	–
Occupation	*F*(4, 5,418) = 4.59, *p* = 0.001	*F*(4, 5,418) = 2.69, *p* = 0.029
Political orientation	*F*(4, 5,418) = 9.30, *p < 0*.001	–
Type of pro-environmental behaviors	*F*(4, 5,418) = 2.60, *p* = 0.034	*F*(4, 5,418) = 11.71, *p* < 0.001
Main environmental concern	*F*(2, 5,418) = 3.24, *p* = 0.039	–

*Profiles most typical for environmentalists.* Environmentalists described as woman, as Asian, working as a cleaner or an office clerk, as politically moderate or liberal, as privately pro-environmentally active, as well as with a mainly global environmental concern were described as most typical for environmentalists.

*Profiles least typical for environmentalists.* Environmentalists described as non-binary, as Hispanic/Latino or Black/African American, working as a corporate CEO, as politically conservative, as radically pro-environmentally active, and with a mainly local environmental concern.

*Strongest self-identification with environmentalists.* Participants identified more strongly with environmentalists that were described as woman, working as a cleaner, and with privately pro-environmental behavior.

*Weakest self-identification with environmentalists.* Participants identified weakest with environmentalists that were described as non-binary, as corporate CEO, and with radical pro-environmental behavior.

**Figure 5:**
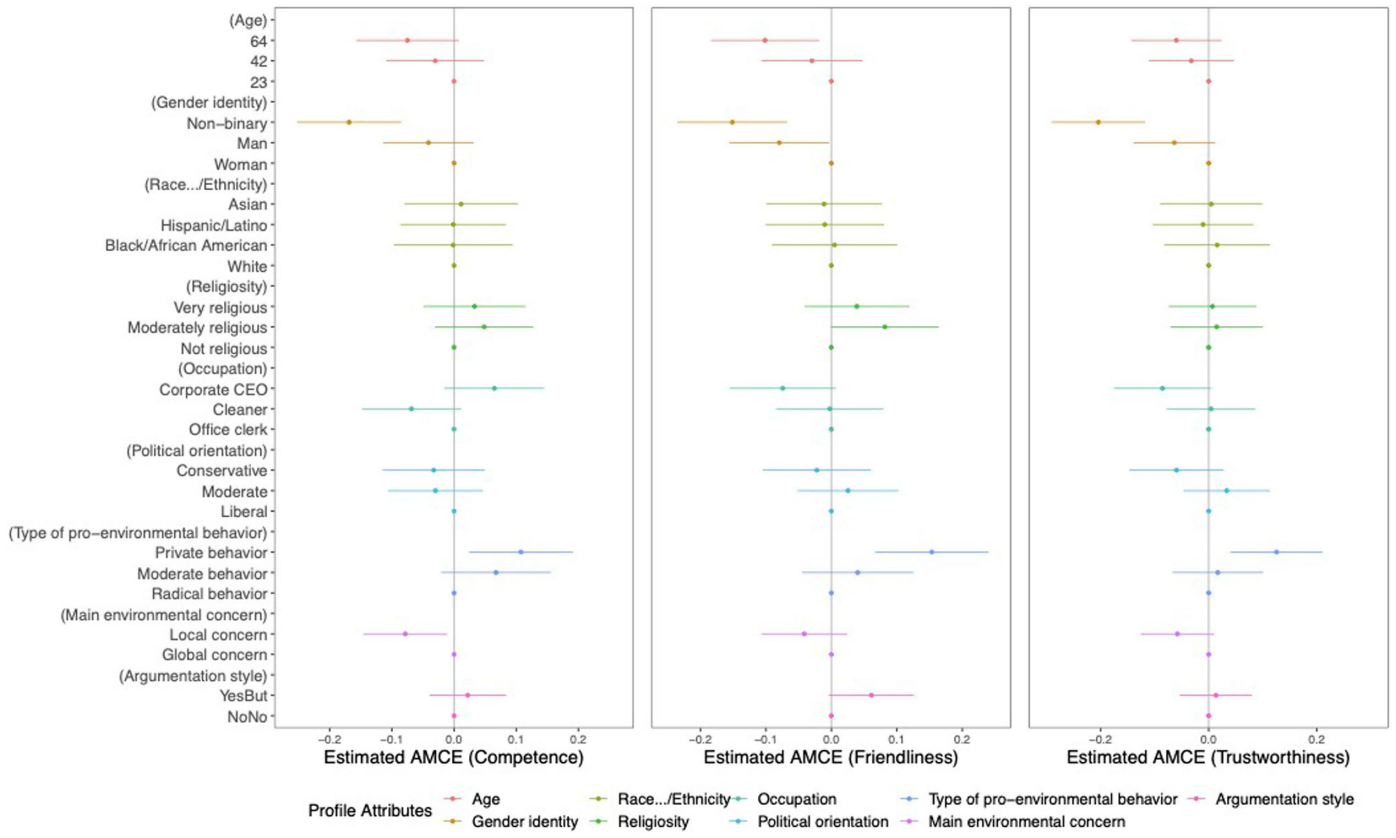
Average marginal component effect estimates for each attribute value on the profile ratings for competence, friendliness, and trustworthiness. The x-axis units indicate the sizes of the AMCEs.

**Figure 6:**
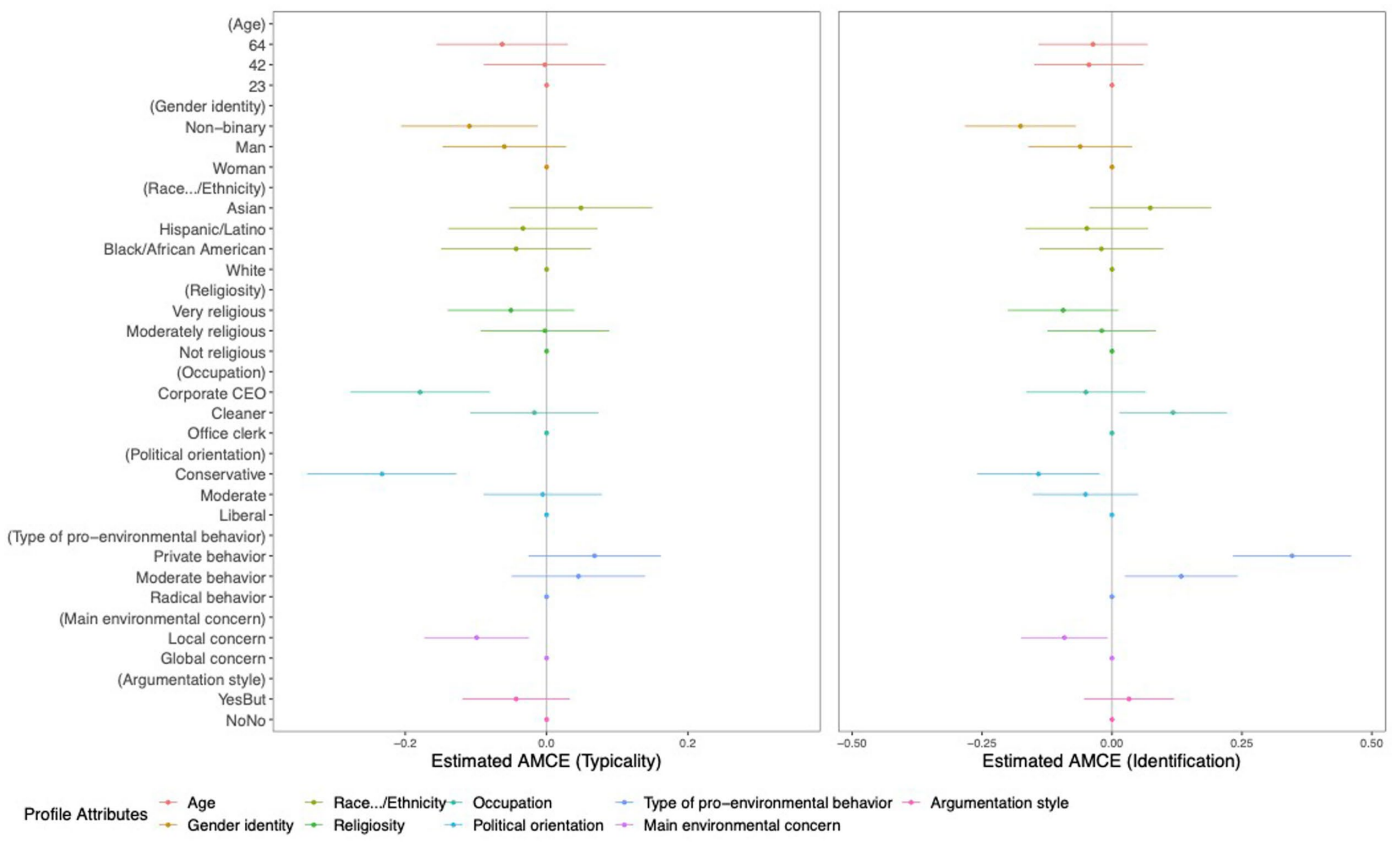
Average marginal component effect estimates for each attribute value on the profile ratings for typicality as environmentalist and self-identification with the profiles. The x-axis units indicate the sizes of the AMCEs.

To compare attribute values, that in previous literature were found to be stereotypic for environmentalists with attribute values that have not been identified as such, we calculated AMCEs. In the following, AMCEs are reported for each profile’s attribute value calculated for the measures of competence, friendliness, and trustworthiness (Figure 5), as well as the profiles’ typicality as environmentalists and the participants’ self-identification with the profiles (Figure 6). Here, the x-axis units indicate the sizes of the AMCEs (not the original scale points). Moreover, the dots represent the estimated AMCEs per attribute value relative to the baseline/reference value (located on the vertical line in the plots), and the bars on either side of these dots are the 95%-Confidence Intervals (CI) for the effects. When the CI does not include the x-axis’ zero point, the effect of the attribute value is significantly different from the reference value. As mentioned earlier, for this study, we chose values that are consistent with the findings of previous stereotype literature as reference values (x-axis’ zero point). Given this context, a significant effect (when the CI not including x-axis zero point) indicates a distinction in participants’ evaluations between stereotype-consistent and stereotype-inconsistent information. The position of the bars indicates whether the attribute value was evaluated more positively or negatively compared to the reference value. The visualized tendencies look similar to the previous plots but differ in the way that the calculated estimates are all relative to the reference value of the given attribute category, thus they can only be compared within that category.

From the calculations (see Supplementary materials) and visualizations (see Figures 5, 6) of the AMCEs, the descriptions of environmentalists with stereotype-inconsistent attribute values shown in Table 5, were perceived significantly more positively and negatively.

**Table 5:** Attribute values of environmentalist profiles evaluated more positively or negatively compared to reference values identified by previous stereotype literature.

Attribute categories	Attribute values evaluated more positively compared to attribute values consistent with stereotype literature	Attribute values evaluated more negatively compared to attribute values consistent with stereotype literature
Age (Reference value: 23 years old)	–	64 years (friendliness, *p* < 0.001)
Gender identity (Reference value: woman)	–	non-binary (competence, *p* < 0.001; friendliness, *p* < 0.001; trustworthiness, *p* < 0.001; typicality, *p* < 0.001; self-identification, *p* < 0.001) and man (friendliness, *p* = 0.041)
Occupation (Reference value: office clerk)	working as cleaner (self-identification, *p* < 0.001)	as corporate CEO (typicality, *p* < 0.001)
Political orientation (Reference value: liberal)	–	conservative (typicality, *p* < 0.001; self-identification, *p* < 0.001)
Type of pro-environmental behaviors (Reference value: radical)	private (competence, *p* = 0.012; friendliness, *p* < 0.001; trustworthiness, *p* = 0.004; self-identification, *p* < 0.001) and moderate (self-identification, *p* < 0.001)	–
Main environmental concern (Reference value: global)	–	local (competence, *p* = 0.023; typicality, *p* < 0.001; self-identification, *p* < 0.001)

## 5. Discussion

The present study sought to broaden our understanding of how public perceives environmentalists as a social category in the US Environmentalists are strongly stereotyped and politicized yet remain an understudied social category. Aiming at identifying the social identity factors that influence public impressions of and self-identification with environmentalists, we wanted to answer (1) which identity factors of fictitious environmentalist profiles led a sample of US residents (1.1) to perceive them as competent, friendly, and trustworthy (1.2) to see them as typical for environmentalists, and (1.3) to self-identify with them. Lastly, building on the work of Bashir et al. (2013) and Stenhouse and Heinrich (2019), we sought to further explore atypical environmentalists (2). Specifically, we analyzed whether fictitious profiles described with attributes that deviate from (vs. align with) established stereotypes would enhance positive impressions of, and self-identification with, these environmentalists.

### 5.1. Effects on perceptions of the profiles’ competence, warmth (friendliness), and morality (trustworthiness)

Overall, our results correspond with our expectations and prior stereotype literature (e.g., Stereotype Content Model; Fiske et al., 2002). For example (H1.1), environmentalist profiles ascribed as women were generally perceived as friendlier and more trustworthy and competent than the other gender identities. However, this is contrary to the findings of Fiske et al. (2002), where men are usually perceived as more competent than women. In light of the SCM quadrants (Fiske et al., 2002), our study reveals admiration for environmentalist women. It is essential to note that a direct comparison of our findings with the SCM’s observations on women is constrained because of the differing measurement methods and the environmental focus of our profile descriptions. Therefore, more research is needed in applying the SCM. By contrast, non-binary profiles were rated the lowest among all three stereotype dimensions. Bearing in mind that the social concept of non-binary gender identity is relatively new (Matsuno and Budge, 2017), our findings could be explained through participants perceiving non-binary environmentalists as unconventional and eccentric. Although eccentricity had been found as a typical trait of environmentalists (Bashir et al., 2013), combining two already unconventional and stereotyped identity dimensions, namely environmentalists and non-binary gender, seemed to have elicited the least positive impressions among participants. Broadening the knowledge in this field, Stenhouse and Heinrich (2019) investigated the mediating role of perceiving environmental activists as eccentric, militant, and friendly; they, too, used a conjoint design and found eccentricity to be least important in increasing the attraction to activists. Our results may align with Stenhouse and Heinrich’s findings as the unconventional non-binary profiles did not improve our participants’ impressions.

Profiles with the high-status occupation as corporate CEO were rated as most competent compared to working as cleaners and office clerks, but not as friendly or trustworthy. These findings correspond to previous research showing that higher status levels predicted higher competence, but competition predicted lower warmth/friendliness (Fiske et al., 2002). In another instance, Fiske and Dupree (2014) found similar effects regarding climate scientists. For a communicator to effectively capture attention and establish credibility, they must not only display expertise (e.g., competence) but also be perceived as both warm and trustworthy (see also Castro and Rosa, 2023). In light of these findings and when applied to our study, the environmentalist profile that most closely resembled a credible communicator was that of a female office clerk.

Our survey participants viewed young environmentalists as friendly compared to older environmentalists. In contrast to previous literature (Fiske et al., 2002), our participants tended to rate young environmentalists overall more positively than older ones. More specifically, profiles of older environmentalists were perceived as neither friendly nor competent. This perception aligns with the SCM by Fiske et al. (2002), where groups viewed as low in both warmth and competence elicit feelings of contempt. Further, considering the average age of participants was *M* = 34.26, this observed bias may be rooted in potential intergenerational tensions, reflecting prejudices often held by younger individuals toward their elder counterparts, as posited by North and Fiske (2012, 2013). Younger people evaluate older adults low on the dimensions of warmth and competence because they may see them as a passive social group (North and Fiske, 2012). Such perceptions might be reinforced through ingroup favoritism (Brewer, 2007). Further empirical investigations are required to address any of these possible explanations.

Highly relevant to the US context is the perception of environmentalists’ political orientation. Respectively, environmentalists with moderate political views were perceived as most trustworthy, and conservatives as least trustworthy. Interestingly, environmentalists with a liberal political orientation were considered most competent but not as friendly compared to political moderates. Overall, participants saw profiles with moderate political views as the most friendly and trustworthy but perceived conservative profiles as the least competent and friendly. As a possible explanation, discussed below, both liberals and conservatives perceived profiles from the ideologically dissimilar group as less friendly and trustworthy. Hence, political moderates who did not pose a threat to either of them were generally preferred (Brandt et al., 2014).

In the US context, race and ethnicity are also particularly relevant: However, our findings did not align with our initial predictions. Contrary to our expectations based on Fiske et al. (2002), White profiles were not perceived as the friendliest and most competent, and Asian profiles were not viewed as solely competent without warmth. Instead, our participants rated profiles based on various racial and ethnic characteristics. Their perceptions in terms of competence, friendliness, and trustworthiness were consistent across these profiles. Nevertheless, also here we need to highlight that Fiske et al. (2002) assessed general perceptions whereas our results specifically refer to how environmentalists are perceived. Further research is needed to replicate our results and to directly compare them with Fiske et al. (2002) findings. Nevertheless, our results could have been influenced by the current debate on systemic racism, the Black Lives Matter movement, and ongoing social tensions in the United States, shifting toward more neutral perceptions across different racial and ethnic groups (Sawyer and Gampa, 2018). Although contrary to our expectations, these results offer hope for actual social change and societal improvements in the United States.

More in line with prior research (Castro et al., 2017; Klas et al., 2019; Castro and Rosa, 2023), survey participants perceived environmentalists with private pro-environmental behaviors as most friendly and trustworthy. But those with moderate pro-environmental behaviors were still perceived more positively than those with radical ones. This finding could be explained by the fact that moderate pro-environmental behavior still had an activist nature (e.g., attribute “Writes political representatives”). Contrary to our expectations and existing literature, our sample perceived environmentalists with radical behaviors as the least competent (Castro et al., 2017; Castro and Rosa 2023). Thus, these findings suggest that the general public tends to disapprove of actions perceived as radical or militant, like demonstrations (Klas et al., 2019). Our findings align with prior research, suggesting that activists with radical discourse may face penalties in terms of perceived warmth, though not necessarily on the competence dimension (Castro et al., 2017; Castro and Rosa, 2023). As further evidence of this, we noted that environmentalists using a conciliatory argumentation style, as opposed to a confrontational one, were viewed as more competent and friendly, albeit without significant differences. In this respect, and based on our results, the lesser environmentalists were described as ostentatious and demonstrative, the more positively they were perceived. Therefore, environmentalists keep facing the *activist dilemma* (Feinberg et al., 2017) in which raising public awareness ends up reducing public support.

In conclusion, our US sample perceived environmentalist profiles most favorably – in terms of competence, friendliness, and trustworthiness, when they were described as young, female, office clerks, with a moderate political orientation, and who engage in pro-environmentally activities primarily on a private level. This aligns with Fiske and Dupree (2014) findings which emphasize credibility as a blend of one’s expertise (i.e., competence) and their genuine motivation to be truthful (i.e., warmth/trustworthiness). Consequently, the traits identified in our study suggest specific characteristics that render an environmentalist more credible in the eyes of the public, marked by perceptions of competence, friendliness, and trustworthiness. This, in turn, can potentially amplify the public’s attention toward them. We recommend further research to investigate this relationship.

### 5.2. Effects on perceptions of the profiles’ typicality as environmentalists

The findings of our study largely supported our expectations regarding which attribute values our sample would identify as typical for environmentalists (H1.2). For example, women were perceived as more typical environmentalists compared to men and non-binary profiles. In terms of race Asians were viewed as the most typical environmentalists, followed by Whites. This aligns with prior studies that link pro-environmental behaviors and heightened environmental concerns to feminine traits (Brough et al., 2016; Swim and Geiger, 2018). This relationship might be attributed to overlapping traits commonly associated with women and environmentalism. Historically, pro-environmentalism has been understood as a caring stance (Rome, 2006) and caretaking has been stereotypically linked to female gender roles (Eagly et al., 2000). Consequently, in comparison to women, men were less frequently perceived as typical environmentalists. However, they were still rated more typical than those with a non-binary gender identity. This could be attributed to the still emerging societal recognition and perceived novelty of non-binary genders (Matsuno and Budge, 2017).

Contrary to our expectations, profiles of Asians individuals were perceived as more typical for environmentalists than those of White individuals. This is surprising given that in previous studies Asian individuals were perceived as being less environmentally concerned than Whites, though they were rated more concerned than other US racial-ethnic minority groups (Pearson et al., 2018). Our results may indicate a shift in the public’s perception of the prevailing image of an environmentalist, thereby broadening our current knowledge. As such, status predicted competence (SCM; Fiske et al., 2002) and the environmentalist identity was seen as related to higher social status (Pearson et al., 2018). Consequently, Asian Americans, who have been stereotyped as highly competent, may be considered more typically aligned with environmentalists. However, such a shift in prototypicality, along with the explanation provided here, should be investigated in future research.

Significant variations in perceptions of typicality emerged concerning environmentalists’ occupations and political orientations. Based on the study by Pearson et al. (2018), we expected profiles of middle-class social status to be perceived as most typical for environmentalists. Accordingly, we found that profiles with the occupation office clerks (representing middle social status) were viewed as most typical. However, contrary to previous literature, people working as cleaners (representing lower social status) were perceived as equally typical. Moreover, corporate CEO profiles were seen as least typical for environmentalists. In this regard, our findings extend previous literature (Pearson et al., 2018) suggesting that occupations indicative of lower social status are not inherently deemed atypical for environmentalists or those environmentally conscious. Furthermore, positions associated with the upper social class jobs, such as corporate CEOs, may be perceived by the public as implausible representations of environmentalists. Similarly, profiles described with conservative political leaning were perceived as less representative of environmentalists in comparison to those with liberal and moderate orientation. These results are in line with previous research indicating that environmentalists are generally associated with left-leaning ideologies or political orientation (Merkley and Stecula, 2018).

Concerning the environmental attributes of the profiles, our results do not support our assumptions that radical pro-environmental behaviors would be perceived as more typical for environmentalists than moderate or private actions (Bashir, 2010; Bashir et al., 2013). Drawing insights from social cognition research on impression formation (Fiske and Neuberg, 1990), it is argued that people’s information processing is a blend of cognitive and motivated. Hence, while our participants showed a clear preference for private pro-environmental actions (see the previous section), these intrinsic motives might have also influenced their perceptions of what is typical.

Participants also viewed environmentalist profiles emphasizing global environmental concerns as more typical compared to those focused on local concerns. Previous research has shown that vulnerable US population segments, particularly People of Color (POC), tend to prioritize local and human-oriented environmental challenges more than the White population (Mohai and Bryant, 1998; Song et al., 2020). Hence, having a mostly White/Caucasians (74.2%) study sample may explain that profiles with local environmental concerns were perceived as less typical for environmentalists. Nevertheless, further analyses would need to be conducted to confirm a possible of the vulnerable and low-status populations’ concerns by more privileged societal groups in the United States (Mohai et al., 2009; Timmons Roberts et al., 2018).

To summarize our findings on the participants’ perception of typicality for environmentalists, the perceptions that corporate CEOs and political conservatives are the least typical environmentalists stand out as some of the most novel results.^5^

### 5.3. Effects on participants’ self-identification with profiles

Concerning H1.3, our results revealed a notably stronger identifications with female environmentalists as opposed to non-binary profiles. Further, participants most strongly identified with environmentalists described as cleaners, to a lesser degree with those labeled as corporate CEOs or office clerks. Additionally, profiles highlighting private pro-environmental behaviors also particularly resonated with our sample.

Since we did not measure other components of self-identification (e.g., self-defining and self-investing as per Leach et al., 2008), we cannot explain in detail how participants’ tendencies to self-identify with certain environmentalist profiles derived. Based on the calculated marginal means, our participants generally did not identify strongly with the presented profiles (see results). This could be due to oversimplified profile descriptions or the measure itself. Future research should delve deeper into nuanced identity facets. However, the level of self-identification with specific environmentalists remains vital in practice, as these profiles may hold greater influence.

### 5.4. Effects of attribute values atypical according to stereotype literature

Building on Bashir et al. (2013) work into atypical environmentalist profiles, we further analyzed the differences between profiles showcasing stereotype-consistent attribute values (e.g., liberal) and those exhibiting stereotype-inconsistent attribute values (e.g., conservative). In our results (H2), we observed both positive (as expected) and negative effects on participants’ judgments of and self-identification with environmentalists.^6^

Profiles of environmentalists with attribute values that deviate from existing stereotypes showed *positive effects* by being perceived as more competent, friendly, and trustworthy, compared to profiles that align with radical (stereotype-consistent) behaviors. Despite literature suggesting radical pro-environmental behaviors as characteristic of environmentalists, our participants predominantly viewed private behaviors as the more typical manifestation (also see in “Effects on perceptions of the profiles’ typicality as environmentalists”). In line with this relationship, participants preferred to self-identify with environmentalists described through private or moderate pro-environmental behaviors rather than through radical ones. Surprisingly, participants’ self-identification with environmentalists was higher when the profiles were described as cleaners instead of as office clerks (stereotype-consistent).

Contrary to what Bashir et al. (2013) suggested, our study also uncovered *negative effects* of stereotype-inconsistent traits on participants’ perceptions. Specifically, attributes portraying environmentalists as non-binary, male, with an age of 64 years, corporate CEOs, political conservatives, or primarily concerned with local environmental issues led to diminished ratings in terms of competence, friendliness, trustworthiness, perceived typicality, and participants’ identification with the profiles. Thus, we conclude that environmentalists are generally preferred when they are only individually or privately active (Castro et al., 2017; Klas et al., 2019) – that is, environmentalists that do not challenge the status quo. Earlier studies have highlighted that activists, often termed as “moral rebels,” are perceived by certain segments of society as a “threat to society” (Hoffarth and Hodson, 2016, p. 40). They are viewed as challengers to the prevailing societal conventions (Lindblom and Jacobsson, 2014), or as entities that threatening people’s positive self-perceptions (Monin et al., 2008). Moreover, our findings suggest that descriptions that are atypical according to stereotype-literature do not necessarily correspond with a better impression of environmentalists.

Our findings may direct future research toward investigating the effects of stereotype-inconsistent environmentalists on impression formation. More specifically, research should investigate how stereotype strength (Allen et al., 2009) and stereotype incongruency (Sekaquaptewa and Espinoza, 2004) influence impression processing.

### 5.5. Limitations and future research

Owing to its novel methodological approach to psychological research, this study presents some limitations. Due to sample size restrictions and statistical power calculations (Stefanelli and Lukac, 2020), our conjoint tables presented a limited number of profile attributes aimed at artificially describing environmentalists. These constraints may have influenced participants’ perception of the described environmentalists in terms of realism. Future research could expand the range and diversity of profile attributes presented in the conjoint tables incorporating a wider array of characteristics that are relevant to environmentalists. This will contribute to a more comprehensive and authentic depiction of environmentalists.

Participant recruitment was accomplished using a combination of sampling approaches, including convenience sampling and paid crowdsourcing. Subsequent group comparisons indicated notable distinctions between the MTurk and convenience sample, raising concerns regarding the potential applicability of our findings to the wider US population. We recommend that future researchers test our hypothesis using a representative sample of US population in order to enhance the generalizability of these findings. Moreover, we need to acknowledge that with the novel methodological approach, we cannot definitively determine whether the observed differences were due to variations in the sample or actual shifts in people’s views. Due to methodological differences that exist between our research and prior studies, we urge readers to wary caution when deriving comparisons.

Given the limited analyses options provided by the Cregg R package (Leeper, 2020), our reporting was restricted to causal interpretations derived from pairwise comparisons via omnibus F-tests and visual plots. Unfortunately, we were unable to incorporate statistical control for covariates or to test the influence of multiple moderating effects and their interactions. In future studies, researchers should consider employing a more comprehensive analysis approach that allows for statistical control of covariates and the examination of multiple moderating effects and their interactions.

## 6. Contributions and concluding remarks

Our study opens new directions regarding impression formation research and the application of conjoint analyses in psychology. For instance, we extend scientific knowledge on identity dimension-specific perceptions of environmentalists (Pearson et al., 2018) in the United States. Specifically for stereotype content literature (Fiske et al., 2002; Leach et al., 2007; Stenhouse and Heinrich, 2019), our findings demonstrate the potential of conjoint analyses by integrating questions on people’s stereotypical judgments via the dimensions of competence, friendliness, and trustworthiness. Moreover, we contribute to previous literature on perceptions of different types of environmentalists (Castro et al., 2017) by mapping the influence of multiple personal attributes (e.g., gender identity, race/ethnicity, political orientation). We contribute further to environmentalist prototype research (Ratliff et al., 2017) by revealing which attribute values were perceived by our sample as most typical. Additionally, we examined the impact of environmentalist descriptions that deviated from established norms in prior literature (Bashir et al., 2013). These level-by-level results provide valuable implications for research on environmental identity (Brick and Lai, 2018).

Our research extends Stenhouse and Heinrich (2019) application of conjoint designs through the application of new attributes and measures. In regard to the ongoing political debates and diminishing public support for environmental causes in the United States, our findings provide environmental movements with valuable input on how to access the public attention through positive and credible images of environmentalists (Fiske and Dupree, 2014) and through intentional message framing customized to the targeted audience (Maxwell and Miller, 2016; Pearson et al., 2018). Using these insights, public portrayals of and interactions with environmentalists can be tailored to align with desired perceptions and target audiences. This offers valuable insights to the environmental movement regarding message source and content to resonate with the targeted audience. Our data indicates that the public tends to favor environmentalists who engage in private sustainable behaviors (Castro et al., 2017). This poses a fundamental problem: while environmentalists are driven to elevate public consciousness about environmental protection and challenge environmental misuse, they face backlash for being perceived as overly aggressive in challenging the status quo. This places them in an “activist’s dilemma” (Feinberg et al., 2017), a paradox where their well-meaning actions inadvertently lead to unintended consequences (Bashir et al., 2013).

In summary, environmental movements need intermediaries who can foster discussions and facilitate consensus within the public sphere. Our findings offer insights on how these intermediaries should be portrayed and perceived to effectively champion pro-environmental causes. We draw from our applied conjoint analysis findings that our participants related most to and judged most positively those environmentalists who were described as women, Asian, working as cleaners, political moderates, with private pro-environmental behaviors, and mainly global environmental concerns. That said, environmentalists occupying a middle ground may be more successful in reaching a diverse range of people and avoid losing further public support. We aspire that, in due time, environmental protection will transcend its polarizing and politicized stature in the United States and that people from all backgrounds will feel included enough to identify (again) with *environmentalists*.

## Data availability statement

The raw data supporting the conclusions of this article will be made available by the authors on request, without undue reservation.

## Ethics statement

The study involving humans was approved by the local ethics committee at Iscte-IUL. The study was conducted in accordance with the local legislation and institutional requirement. The participants provided their written informed consent to participate in this study.

## Author contributions

KK and MR conceived and designed the study. KK adapted all coding, created the questionnaire, collected the data, analyzed the data, and wrote an initial draft based on the results. MR and MO critically revised the draft manuscript and made important changes in content. All authors contributed to the article and approved the submitted version.

## Footnotes

^1^Environmental justice is the fair treatment and meaningful involvement of all people regardless of race, color, national origin, or income, with respect to the development, implementation, and enforcement of environmental laws, regulations, and policies. This goal will be achieved when everyone enjoys the same degree of protection from environmental and health hazards and equal access to the decision-making process to have a healthy environment in which to live, learn, and work (United States Environmental Protection Agency, US EPA 2016).

^2^Further details in Supplementary materials.

^3^See Supplementary materials for further details, the full questionnaire, as well as the HTML and JavaScript coding.

^4^For clarification, *Attributes* refer to the name of features or characteristics that describe the profiles. These attributes consist of *levels* or *values* representing the different choices for each attribute.

^5^From the survey comments, we could comprehend to which extent participants perceived environmentalists as highly unrealistic when described as corporate CEOs and political conservatives.

^6^These discussed findings refer to the significant differences found between attribute values that are consistent vs. inconsistent with previous literature. Moreover, the overall patterns are similar to those addressed in the previous section.

## Supplementary material

The Supplementary material for this article can befound online at: https://www.frontiersin.org/articles/10.3389/fpsyg.2023.1125617 /full#supplementary-material

SUPPLEMENTARY PRESENTATION 1 Further information on conjoint analysis.

SUPPLEMENTARY PRESENTATION 2 Recruitment messages.

SUPPLEMENTARY PRESENTATION 3 Coding of conjoint table in Qualtrics.

SUPPLEMENTARY PRESENTATION 4 Online Qualtrics questionnaire.

SUPPLEMENTARY PRESENTATION 5 Display frequencies-descriptives-correlations.

SUPPLEMENTARY PRESENTATION 6 Further results of the conjoint analyses.
