# Supplementary material for: How different types of environmentalists are perceived: changing perceptions by the feature
Source: Front Psychol. 2023 Nov 9;14:1125617. doi: 10.3389/fpsyg.2023.1125617 (PMC10666641; doi:10.3389/fpsyg.2023.1125617)
Supplement: SUPPLEMENTARY PRESENTATION 1 — Further information on conjoint analysis. [file Presentation_1.pdf]

Further information on conjoint analysis

Assumptions of conjoint analysis

To conduct a conjoint experiment, Hainmueller et al. (2014, pp.8–9, 13, 16) advise to adopt five basic assumptions. First, when analyzing multiple profiles, participants' responses are stable and there are no carryover effects from profile attributes viewed earlier. Second, the position of the profile within the task (e.g., in left or right table column) does not affect the responses. Third, the presented profiles are factually and completely generated at random. This means that each attribute is randomized either conditionally to or completely independent of the other attributes.

Strengths and benefits of the conjoint analysis

The conjoint analysis is a very useful tool to understand preferences / favorability among multidimensional alternatives. The aim of conjoint experiments and analysis is to identify, through descriptive and causal effect interpretations of favorability, which combinations of attributes are most influential on participants' decision and response behavior (Strezhnev et al., 2013). Moreover, conjoint analyses offer several strengths and benefits. For instance, they come closer to real-world behavior than traditional vignettes (Hainmueller et al., 2015) therefore increasing external validity (Hainmueller et al., 2014). They are also time and cost-efficient, estimating the multiple factorial effects with feasible sample sizes and large statistical power that otherwise would involve impractical designs (Hainmueller et al., 2014), while limiting social desirability (Horiuchi et al., 2020).

Statistical analysis of conjoint designs

After the presentation of at least two profile descriptions at a time, participants are asked to select or rate, for example, which article to buy (Kulshreshtha et al., 2019), whom to vote for (Doherty et al., 2019), which job candidate to select (Carey et al., 2020), or which immigrant to accept into a country (Berinsky et al., 2020). These questions represent the dependent variables of the conjoint experiment which need to be minimalistic and shouldn't consist of long scales. They can even just be the choice between one or the other. Moreover, these questions are referred to as *conjoint tasks* and serve as outcome measures to estimate the participants' "preferences" (Hainmueller et al., 2014) or "favorability" (Leeper et al., 2020) through the marginal effect of each attribute value, instead of the whole profile (Stenhouse and

Heinrich, 2019). *Preferences* and *favorability* refer to a statement of support for the chosen or to be rated object or person (Leeper et al., 2020).

The marginal effects indicating favorability are calculated through values known as the Average Marginal Component Effect or *AMCE* (Hainmueller et al., 2014) and Marginal Means or *MMs* (Leeper et al., 2020). “They measure the degree to which a given value of a conjoint profile feature increases, or decreases, participants’ support for the overall profile, averaging across all participants and other features” (Leeper et al., 2020, p.1). Characteristic for conjoint analyses, both values provide descriptive and causal effect interpretations presented as absolute or relative favorability (Leeper et al., 2020).
