# Supplementary material for: How different types of environmentalists are perceived: changing perceptions by the feature
Source: Front Psychol. 2023 Nov 9;14:1125617. doi: 10.3389/fpsyg.2023.1125617 (PMC10666641; doi:10.3389/fpsyg.2023.1125617)
Supplement: SUPPLEMENTARY PRESENTATION 2 — Recruitment messages. [file Presentation_2.pdf]

Recruitment messages

Figure 11

Recruitment text published in social media (i.e., Facebook, Instagram, and LinkedIn)

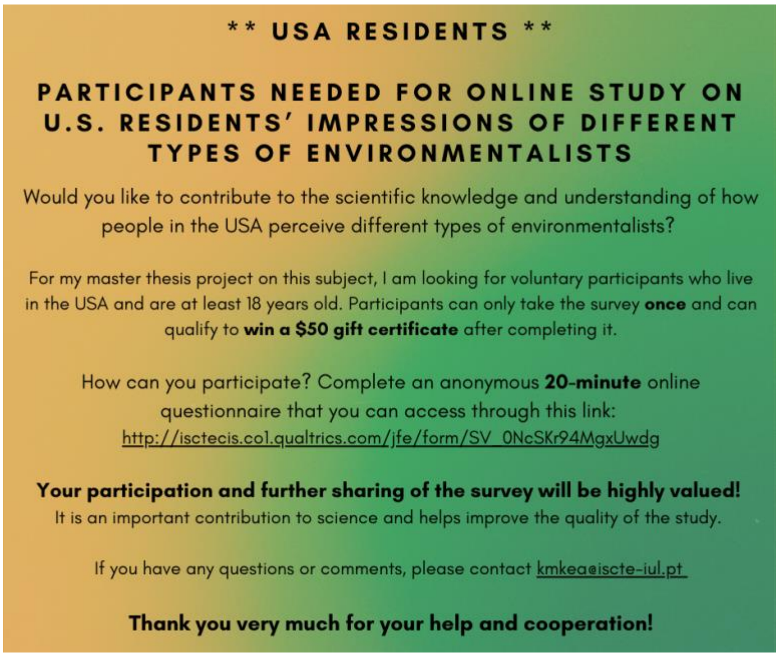

Figure 12

Recruitment text published on Amazon MTurk

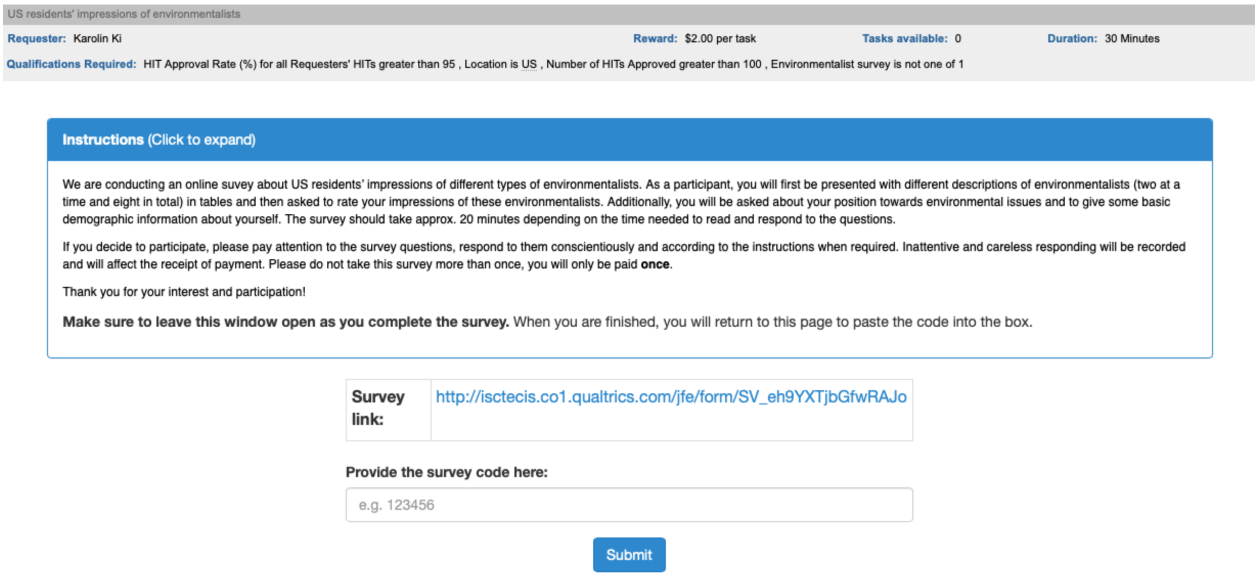

US residents' impressions of environmentalists

Requester: Karolin Ki **Reward:** $2.00 per task **Tasks available:** 0 **Duration:** 30 Minutes

Qualifications Required: HIT Approval Rate (%) for all Requesters' HITs greater than 95 , Location is US , Number of HITs Approved greater than 100 , Environmentalist survey is not one of 1

Instructions (Click to expand)

We are conducting an online suvey about US residents' impressions of different types of environmentalists. As a participant, you will first be presented with different descriptions of environmentalists (two at a time and eight in total) in tables and then asked to rate your impressions of these environmentalists. Additionally, you will be asked about your position towards environmental issues and to give some basic demographic information about yourself. The survey should take approx. 20 minutes depending on the time needed to read and respond to the questions.

If you decide to participate, please pay attention to the survey questions, respond to them conscientiously and according to the instructions when required. Inattentive and careless responding will be recorded and will affect the receipt of payment. Please do not take this survey more than once, you will only be paid **once**.

Thank you for your interest and participation!

Make sure to leave this window open as you complete the survey. When you are finished, you will return to this page to paste the code into the box.

**Survey link:**	http://isctecis.co1.qualtrics.com/jfe/form/SV_eh9YXTjbGfwRAJo

Provide the survey code here:

e.g. 123456

Submit

MTurk worker qualifications: 95% HIT approval rate, Location in U.S., Number of HITs approved >100, and hadn't participated before (integrated in the HIT)

MTurk control checks: Captcha check, attention checks, page timing, total duration, MTurk worker ID, and unique randomized code (integrated in Qualtrics survey)
