# Supplementary material for: How different types of environmentalists are perceived: changing perceptions by the feature
Source: Front Psychol. 2023 Nov 9;14:1125617. doi: 10.3389/fpsyg.2023.1125617 (PMC10666641; doi:10.3389/fpsyg.2023.1125617)
Supplement: SUPPLEMENTARY PRESENTATION 3 — Coding of conjoint table in Qualtrics. [file Presentation_3.pdf]

Coding of conjoint table in Qualtrics

Figure 13

Snapshot of HTML code of the conjoint table

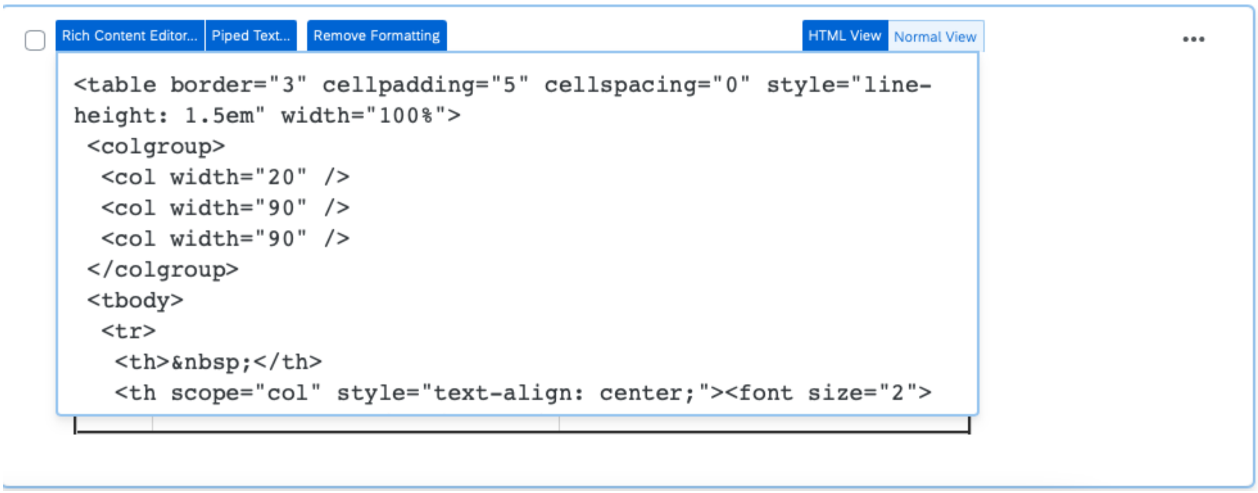


```
<table border="3" cellpadding="5" cellspacing="0" style="line-
height: 1.5em" width="100%">
 <colgroup>
  <col width="20" />
  <col width="90" />
  <col width="90" />
 </colgroup>
 <tbody>
  <tr>
   <th> </th>
   <th scope="col" style="text-align: center;"><font size="2">
```

Note. Feel free to contact author for access to full code.

Figure 14

Snapshot of JavaScript edited in Qualtrics that is responsible for saving the presented profiles with their exact attribute order and values, which are later exported to a .csv file.

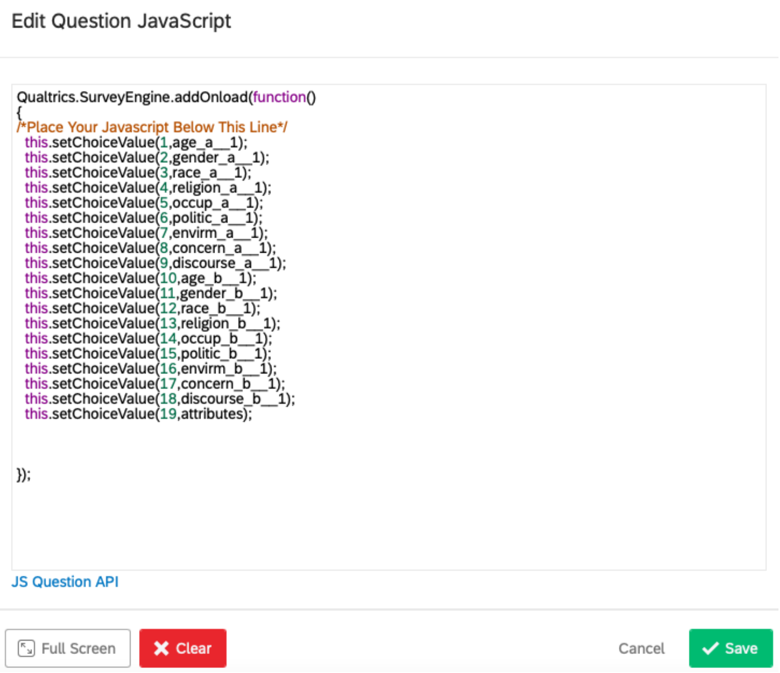


```
Qualtrics.SurveyEngine.addOnload(function()
{
/*Place Your Javascript Below This Line*/
  this.setChoiceValue(1,age_a__1);
  this.setChoiceValue(2,gender_a__1);
  this.setChoiceValue(3,race_a__1);
  this.setChoiceValue(4,religion_a__1);
  this.setChoiceValue(5,occup_a__1);
  this.setChoiceValue(6,politic_a__1);
  this.setChoiceValue(7,envirm_a__1);
  this.setChoiceValue(8,concern_a__1);
  this.setChoiceValue(9,discourse_a__1);
  this.setChoiceValue(10,age_b__1);
  this.setChoiceValue(11,gender_b__1);
  this.setChoiceValue(12,race_b__1);
  this.setChoiceValue(13,religion_b__1);
  this.setChoiceValue(14,occup_b__1);
  this.setChoiceValue(15,politic_b__1);
  this.setChoiceValue(16,envirm_b__1);
  this.setChoiceValue(17,concern_b__1);
  this.setChoiceValue(18,discourse_b__1);
  this.setChoiceValue(19,attributes);


});
```

Note. Feel free to contact author for access to full code.
