# Supplementary material for: How different types of environmentalists are perceived: changing perceptions by the feature
Source: Front Psychol. 2023 Nov 9;14:1125617. doi: 10.3389/fpsyg.2023.1125617 (PMC10666641; doi:10.3389/fpsyg.2023.1125617)
Supplement: SUPPLEMENTARY PRESENTATION 4 — Online Qualtrics questionnaire. [file Presentation_4.pdf]

Online Qualtrics questionnaire

Figure 15

Welcome message and informed consent

iscte INSTITUTO UNIVERSITÁRIO DE LISBOA

Welcome & Informed consent

Welcome and thank you for participating in this survey!

Everyone has different images when thinking of environmentalists.

For the purpose of this study, the term ***environmentalist*** is used broadly to refer to a person who cares and is concerned with or advocates for the protection and improvement of the(ir) environment through different means.

This may include conservationists, preservationists, ecologists, nature-lovers, or otherwise environmentally-minded people.

The present survey is conducted in the context of a master's thesis project underway at ISCTE - Instituto Universitário de Lisboa, Portugal and concerns the study of U.S. residents' impressions of different types of environmentalists.

The research project is carried out by the student Karolin Kibele (kmkea@iscte-iul.pt), who can be contacted for any questions or comments. Please note that only participants who are at least 18 years old and are currently a resident of the United States of America are eligible to participate in this study.

Your participation in the survey will be highly valued as it will contribute to the increase of existing knowledge in this field of research. The survey consists of an online questionnaire in which you will first be presented with different descriptions of environmentalists (two at a time and eight in total) in tables and then asked to rate your impressions of these environmentalists. Additionally, you will be asked about your position towards environmental issues and to give some basic demographic information about yourself.

The entire survey should last approximately **20 minutes** and there are no expected risks associated with participating.

There are no direct benefits to your participation in this study. But at the end of the survey, you can qualify to win a $50 gift certificate.

Your participation is strictly voluntary, and you can stop the survey at any time without any justification. Furthermore, your participation is anonymous; you will never be asked to identify yourself and your responses will be treated strictly confidential.

No answer will be analyzed or reported individually, and no IP addresses used to complete the survey will be saved. The collected data are intended merely for statistical processing and will be stored at a securely and retained indefinitely.

Figure 16

Informed consent, eligibility, and demographical questions

Would you like to participate in this study?

By selecting **YES**, you confirm that you have read and understood the information provided above and that you consent to participate as a subject in this study. You acknowledge that for any questions regarding the research project you can contact the principal investigator Karolin Kibele (kmkea@iscte-iul.pt) or express comments and concerns at the end of the survey.

By selecting **NO,** you choose not to participate, and the survey will end immediately.

Please select one of the following options.

- **YES**, proceed to study
- **NO**, end survey

Please note that only U.S. residents that are at least 18 years old can complete this survey.

Are you at least 18 years old?

- Yes
- No

Are you currently a resident of or living in the United States of America?

- Yes
- No

Not eligible and end of survey

You are not eligible to participate in this survey. Thank you for your time and consideration.

Quota demographics

To begin with, please answer the following demographic questions honestly.

If you were asked to use one of the following three categories for your social class, which would you say you belong to?

- Upper class
- Middle class
- Lower class

Which term(s) best describes your racial / ethnic origin? (You can select multiple answers)

- White / Caucasian
- Black or African American
- Hispanic or Latino
- Asian or Asian American
- Middle Eastern
- American Indian or Alaska Native
- Native Hawaiian or Pacific Islander
- 2 or more of these. Please, write below which
- Other. Please, write below which

Figure 17

Example of (one of four) conjoint table and task

Study introduction

For the next part of the survey, **please imagine you were meeting the environmentalists described to you in the following**.
As explained earlier, you will see two profile descriptions at a time in form of a table.

For each pair, please take your time and read the described details very carefully.

Please pay attention, there will be attention checks which you have to answer correctly.

Conjoint 1

As mentioned earlier, the term ***environmentalist*** is used broadly to refer to a person who cares and is concerned with or advocates the protection and improvement of the(ir) environment through different means. This may include conservationists, preservationists, ecologists, nature-lovers, or otherwise environmentally minded people.

	Environmentalist A	Environmentalist B
Main environmental concern	Global environmental problems (e.g., climate change, depletion of the ozone layer, destruction of wildlife and forests, droughts & floodings)	Global environmental problems (e.g., climate change, depletion of the ozone layer, destruction of wildlife and forests, droughts & floodings)
Age	64	42
Type of pro-environmental behavior	Writes political representatives on environmental regulation issues. Signs petitions on environmental protection. Promotes pro-environmental behaviors to family and friends and by sharing information through social media.	Actively involved in environmental protection groups. Frequently participates in demonstrations, civil disobedience, or other direct actions aiming to influence environmental politics.
Occupation	Cleaner	Corporate CEO
Religiosity	Moderately religious	Not religious
Race/Ethnicity	Black/African American	Hispanic/Latinx
Argumentation style	We are already doing something positive, but we also need changes from large economic groups.	We are already doing something positive, but we also need changes from large economic groups.
Gender	Woman	Man
Political orientation	Moderate	Moderate

When we meet a new person we tend to form a first impression, even if we do not have much information about them.

Now, please rate intuitively the following impressions you have of each described environmentalist as if they were real individuals you are meeting in person.

There are no right or wrong answers (except for the attention checks).

How much do you agree or disagree with each of these statements?
Environmentalist A is...

	Strongly disagree	Disagree	Somewhat disagree	Neither agree nor disagree	Somewhat agree	Agree	Strongly agree
Competent	○	○	○	○	○	○	○
Friendly	○	○	○	○	○	○	○
Trustworthy	○	○	○	○	○	○	○

How much do you agree or disagree with each of these statements?
Environmentalist B is...

	Strongly disagree	Disagree	Somewhat disagree	Neither agree nor disagree	Somewhat agree	Agree	Strongly agree
Competent	○	○	○	○	○	○	○
Friendly	○	○	○	○	○	○	○
Trustworthy	○	○	○	○	○	○	○

How much do you agree or disagree with each of these statements?

	Strongly disagree	Disagree	Somewhat disagree	Neither agree nor disagree	Somewhat agree	Agree	Strongly agree
**Person A** is a typical environmentalist.	○	○	○	○	○	○	○
**Person B** is a typical environmentalist.	○	○	○	○	○	○	○

How much do you agree or disagree with each of these statements?

	Strongly disagree	Disagree	Somewhat disagree	Neither agree nor disagree	Somewhat agree	Agree	Strongly agree
I can identify myself with **Environmentalist A**.	○	○	○	○	○	○	○
I can identify myself with **Environmentalist B**.	○	○	○	○	○	○	○

Figure 18

Questions regarding participants' environmental standpoints

Environmental position

How much do you agree or disagree with this statement?

I would describe myself as an environmentalist.

Strongly disagree	Disagree	Somewhat disagree	Neither agree nor disagree	Somewhat agree	Agree	Strongly agree
○	○	○	○	○	○	○

On a scale from 0 to 10, how concerned are you personally about environmental problems?
(0 indicates that you are not concerned at all, and 10 that you are extremely concerned)

Not concerned at all — Extremely concerned

0 1 2 3 4 5 6 7 8 9 10

Which pro-environmental behaviors, if any, have you integrated in your life? (You can select multiple answers)

- ☐ Actively involved in environmental protection groups.
- ☐ Participating in demonstrations, civil disobedience, or other direct actions aiming to influence environmental politics.
- ☐ Promoting pro-environmental behaviors and sharing information with family, friends, and/or through social media.
- ☐ Voting for pro-environmental candidates, writing political representatives on environmental issues or signing petitions on environmental protection.
- ☐ Preferring to purchase environmentally friendly goods, such as local organic food, or recycled products.
- ☐ Separating garbage at home and using (natural) resources responsibly, like avoids wasting food, energy or water, or drives less by car.
- ☐ None of these

Here is a list of some environmental issues.
Which problems, if any, do you think are more important and urgent today?

- ○ Too much trash and noise in neighborhoods. Polluted tap water and air, as well as the proximity of polluting industrial sites. Lack of access to natural areas or public resources (e.g., grocery stores).
- ○ Climate change and the depletion of the ozone layer. The destruction of wildlife and forests, as well as droughts and floodings across the world.
- ○ None of these

How much do you agree or disagree with this statement?

	Strongly disagree	Disagree	Somewhat disagree	Neither agree nor disagree	Somewhat agree	Agree	Strongly agree
This is an attention check. Please click "Disagree"	○	○	○	○	○	○	○

Figure 19

Questions regarding participants' socio-demographic data

Demographics

Please respond to the following demographic questions accurately.
If a question causes you discomfort, feel free to omit answering that question.

What is your age?

[ ]

Which gender do you identify with?

- ○ Woman
- ○ Man
- ○ [ ] I identify my gender as (please specify)

Which religion do you affiliate with?

- ○ Christian
- ○ Jewish
- ○ Muslim
- ○ Buddhist
- ○ Hindu
- ○ Atheist or agnostic
- ○ None
- ○ [ ] Other. Please, write below which

As how religious/spiritual would you describe yourself?

	1	2	3	4	5	6	7	
Not religious at all	○	○	○	○	○	○	○	Very religious

What is the highest degree or level of school you have completed?

- ○ Did not finish high school
- ○ High school degree or equivalent
- ○ Trade / Technical / Vocational training
- ○ 2-year degree / Associate's degree
- ○ 4-year degree / Bachelor's degree
- ○ Professional / Master's degree
- ○ Doctoral degree

What is your yearly household income?

- ○ Less than $10,000
- ○ $10,000 - $29,999
- ○ $30,000 - $49,999
- ○ $50,000 - $69,999
- ○ $70,000 - $89,999
- ○ $90,000 - $119,999
- ○ $120,000 - $149,999
- ○ $150,000 - $179,999
- ○ $180,000 - $209,999
- ○ More than $210,000

In general, how liberal or conservative are you?
Please rate on a scale from 1 (strongly liberal) to 7 (strongly conservative)

	1	2	3	4	5	6	7	
Strongly liberal	○	○	○	○	○	○	○	Strongly conservative

Figure 20

Questions that were optional for participants to respond to

Optional block

You are almost at the end of the survey!
The next two questions are optional, if you do not have time to respond to these please proceed to the end of the survey.

In which region in the USA do you live?

How much do you agree or disagree with each of these statement?

	Strongly disagree	Disagree	Somewhat disagree	Neither agree nor disagree	Somewhat agree	Agree	Strongly agree
I would describe myself as a global citizen	○	○	○	○	○	○	○

Please select (if any) the political, religious or other community organizations or groups that you could identify with and would advocate for. You can chose multiple or none.

- ☐ Faith-based
- ☐ Environmental protection
- ☐ Peace
- ☐ Student/alumni association
- ☐ Political action
- ☐ Local community
- ☐ Refugee, ethnic minority or immigrant
- ☐ Professional association
- ☐ Media
- ☐ Feminist / women's rights
- ☐ Development or international aid
- ☐ Union
- ☐ Racial justice
- ☐ Reproductive rights
- ☐ Animal protection
- ☐ Human rights
- ☐ LGBTQ+
- ☐ Other. Please write which

Figure 21

Debriefing, explanation of the research, and option to leave comments as well as sign up for the study incentive

Debriefing & Explanation of the research

Thank you for your time and for having participated in this survey!
Your participation is highly valued as it contributes to the increase of scientific knowledge in this field of research.

Please note: We kindly ask you not to disclose this information about the research procedures and purpose to anyone who might participate in this study as this could affect the results of the study.

As indicated at the onset of your participation, this master thesis project is about U.S. residents' impressions of different types of environmentalists.

More specifically, the study aims to understand how, and which characteristics of environmentalists influence how moral, warm and competent they are perceived by people living in the USA, as well as how typical they are as environmentalists and how much participants can identify with the presented profiles. Furthermore, the extent to which participants' own socio-demographic data and identification as environmentalists influence these impressions are examined.

The overall goal of the study is to extend the knowledge on public impressions of environmentalists, as well as which characteristics increase positive associations and identification of a diverse range of U.S. residents with environmentalists. We hope to provide the scientific community and environmental movements with an improved understanding of diversity dimensions that need to be addressed to increase member diversity and public support.

In the context of your participation, the descriptions of environmentalists you were presented with were fully randomized and every participant was presented with a different combination of profile characteristics. Unfortunately, we were only able to include a limited amount of characteristics due to the given research method. This has led to the lack of representation of other important social groups active in environmental protection.

Please remember that the following contact details can be used for any questions you may have, comments you wish to share, or to indicate your interest in receiving information about the outcomes and conclusions of the study: Karolin Kibele, email: kmkea@iscte-iul.pt.

If you want to share somthing you find important, a question that made you feel uncomfortable or that wasn't asked in the survey, please mention them here:

[ ]

Incentive

Now you have the option to sign up to participate in a drawing to win a **$50 gift certificate**.

If you choose to participate, you will be redirected to another survey. There you will be asked to indicate your contact information without being associated with your answers from this survey.

- ○ **Yes**, I want to participate
- ○ **No**, I do not want to participate

Powered by Qualtrics
