# Supplementary material for: How different types of environmentalists are perceived: changing perceptions by the feature
Source: Front Psychol. 2023 Nov 9;14:1125617. doi: 10.3389/fpsyg.2023.1125617 (PMC10666641; doi:10.3389/fpsyg.2023.1125617)
Supplement: SUPPLEMENTARY PRESENTATION 5 — Display frequencies-descriptives-correlations. [file Presentation_5.pdf]

Display frequencies, descriptives, and correlations

Figure 22

Display frequencies of conjoint attribute values in conjoint table

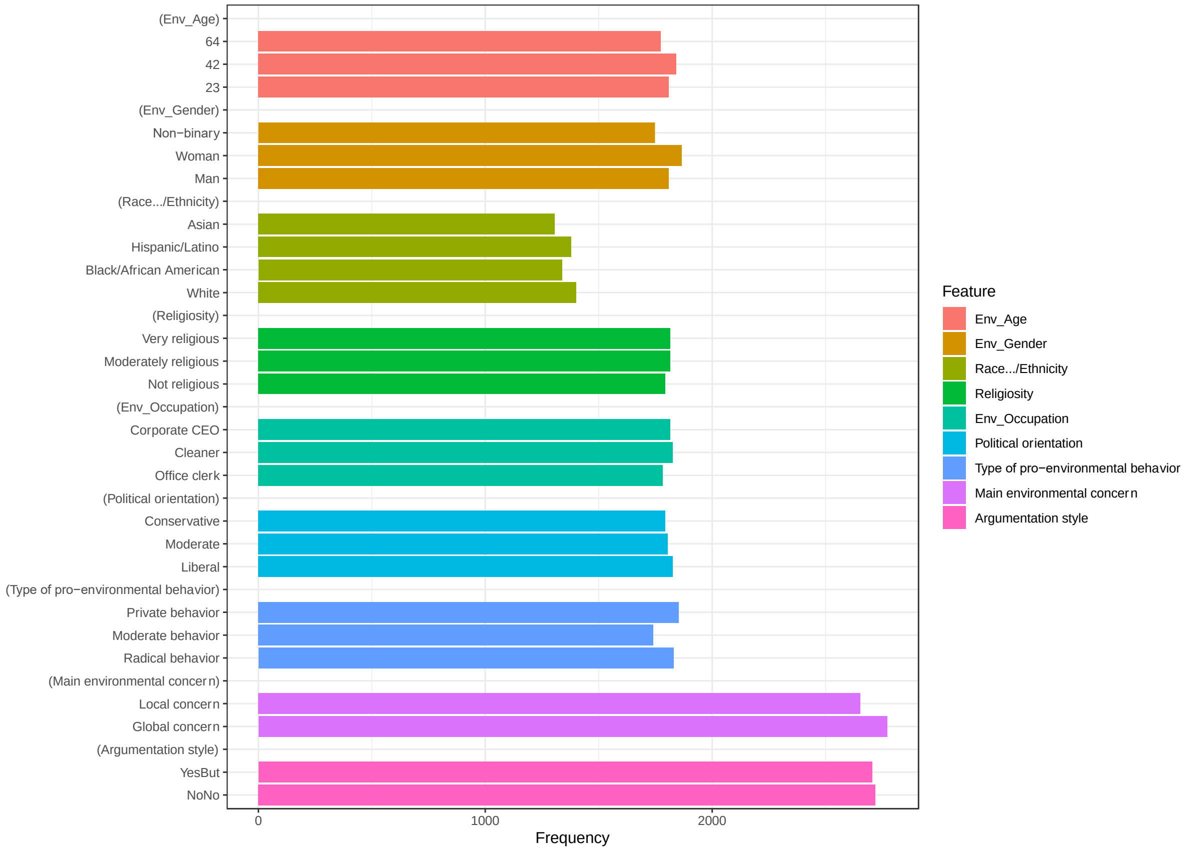

Table 4

Descriptive statistics and Correlations for the variables assessed in the conjoint experiment (DVs and subgroup variables)

Variables	*M*	*SD*	1	2	3	4	5	6	7	8	9	10	11	12	13	14	15	16	17
1 Age	34.26	12.15	-																
2 Gender identity	-	-	.02	-															
3 Self-assessed social class	-	-	.01	-.11***	-														
4 Race/Ethnicity	-	-	-.09***	.04**	-.03*	-													
5 Majority vs. Minority	-	-	-.08***	.07***	-.04**	.77**	-												
6 Religiosity/ Spirituality	3.89	2.20	.01	-.16***	.14***	-.01	.03	-											
7 Political Orientation	3.65	1.86	.00	-.10***	.10***	-.00	.04**	.41***	-										
8 Yearly Household income	-	-	.14***	-.08***	.39***	-.02	-.08***	.02	.03*	-									
9 Sample source	-	-	.25***	.21***	-.20***	.00	.09***	.03*	.07***	-.19***	-								
10 Self-identification as environmentalist	5.16	1.30	.02	-.03*	.14***	-.09***	-.10***	.16***	-.10***	.04*	-.03*	-							
11 Level of environmental concern	7.28	1.98	.07***	-.00	.11***	-.02	-.04**	.02	-.28***	.05***	-.07***	.67**	-						

12 Level of pro-environmental behaviors	7.61	4.86	.02	-.04**	.11***	-.03*	-.04**	.09***	-.25***	.05***	-.11***	.54**	.52**	-					
13 Main environmental concern	-	-	.02	.11***	.02	-.03	-.08***	-.21***	-.26***	.04**	.00	-.04**	.05***	.01	-				
14 Competence	5.35	1.23	-.02	-.04*	.00	.00	.00	-.00	-.08***	.01	.04**	.21***	.247	.18***	-.02	-			
15 Friendliness	5.20	1.23	-.05***	-.06***	.03*	.01	.04**	.07***	.00	-.01	.08***	.21***	.19***	.13***	-.08***	.60**	-		
16 Trustworthiness	5.22	1.25	-.02	-.07***	.05***	.02	.06***	.08***	.00	.01	.08***	.23***	.20***	.13***	-.09***	.61**	.74**	-	
17 Typicality	5.01	1.38	-.04**	-.06***	.05***	.06***	.09***	.13***	.10***	-.02	.04**	.17***	.12***	.09***	-.094	.42***	.45***	.48**	-
18 Identification	4.61	1.58	-.05***	-.04**	.12***	-.00	.05***	.15***	.03*	-.02	.01	.37***	.29***	.22***	-.10***	.43***	.50**	.52**	.54**

Note. *M*=Means, *SD*=Standard deviation. Correlation coefficients. *p < .05. **p < .01 ***p < .001
