# Supplementary material for: How different types of environmentalists are perceived: changing perceptions by the feature
Source: Front Psychol. 2023 Nov 9;14:1125617. doi: 10.3389/fpsyg.2023.1125617 (PMC10666641; doi:10.3389/fpsyg.2023.1125617)

Further results of the conjoint analyses

Assumptions testing in this study

Applying the checks recommended by Hainmueller et al. (2014) showed that all three assumptions were met. First, there were no differences if environmentalists' profiles were presented on the left or right side of the conjoint table, indicating that there were no carry-over effects (Competence, *p*=.313; Friendliness, *p*=.138; Trustworthiness, *p*=.360; Typicality, *p*=.546; Identification, *p*=.430). Second, no differences were found between the presented tables, indicating that there were no table order effects (Competence, *p*=.770; Friendliness, *p*=.764; Trustworthiness, *p*=.310; Typicality, *p*=.954; Identification, *p*=.952). Third, the last assumption was guaranteed through the randomization code generated for the conjoint experiment in Qualtrics. Nevertheless, balance checks of the participants' variables and display frequencies of the attribute values (see supplementary material on display frequencies) confirmed the assumption was met.

Estimates (visualized in the plots integrated in study)

Figure 23

Marginal Mean calculations from R for the profile ratings on environmentalists' Competence

	outcome	statistic	feature	level	estimate	std.error	z	p	lower	upper
1	Rate_Competence	mm	Age	23	5.378662	0.04076150	131.9545	0	5.298771	5.458553
2	Rate_Competence	mm	Age	42	5.357763	0.04303826	124.4884	0	5.273410	5.442117
3	Rate_Competence	mm	Age	64	5.313029	0.04337379	122.4940	0	5.228018	5.398040
4	Rate_Competence	mm	Gender identity	Man	5.376451	0.04077368	131.8608	0	5.296536	5.456366
5	Rate_Competence	mm	Gender identity	Woman	5.417247	0.04054369	133.6150	0	5.337783	5.496711
6	Rate_Competence	mm	Gender identity	Non-binary	5.251144	0.04466433	117.5691	0	5.163604	5.338685
7	Rate_Competence	mm	Race/Ethnicity	White	5.342612	0.04263035	125.3241	0	5.259058	5.426166
8	Rate_Competence	mm	Race/Ethnicity	Black/African American	5.351271	0.04715871	113.4737	0	5.258841	5.443700
9	Rate_Competence	mm	Race/Ethnicity	Hispanic/Latino	5.351234	0.04321433	123.8301	0	5.266535	5.435932
10	Rate_Competence	mm	Race/Ethnicity	Asian	5.355777	0.04717806	113.5226	0	5.263309	5.448244
11	Rate_Competence	mm	Religiosity	Not religious	5.320134	0.04483609	118.6574	0	5.232257	5.408011
12	Rate_Competence	mm	Religiosity	Moderately religious	5.371901	0.04102873	130.9302	0	5.291486	5.452316
13	Rate_Competence	mm	Religiosity	Very religious	5.357930	0.04077493	131.4025	0	5.278012	5.437847
14	Rate_Competence	mm	Occupation	Office clerk	5.350730	0.03909970	136.8484	0	5.274096	5.427364
15	Rate_Competence	mm	Occupation	Cleaner	5.282978	0.04405745	119.9111	0	5.196627	5.369329
16	Rate_Competence	mm	Occupation	Corporate CEO	5.417080	0.04446108	121.8387	0	5.329938	5.504222
17	Rate_Competence	mm	Political orientation	Liberal	5.370208	0.04083074	131.5236	0	5.290181	5.450235
18	Rate_Competence	mm	Political orientation	Moderate	5.343490	0.04149142	128.7854	0	5.262169	5.424812
19	Rate_Competence	mm	Political orientation	Conservative	5.336308	0.04424976	120.5952	0	5.249580	5.423036
20	Rate_Competence	mm	Type of pro-environmental behavior	Radical behavior	5.292896	0.04465393	118.5315	0	5.205376	5.380416
21	Rate_Competence	mm	Type of pro-environmental behavior	Moderate behavior	5.359563	0.04246865	126.2005	0	5.276326	5.442800
22	Rate_Competence	mm	Type of pro-environmental behavior	Private behavior	5.397733	0.04166200	129.5601	0	5.316077	5.479389
23	Rate_Competence	mm	Main environmental concern	Global concern	5.390112	0.03830064	140.7316	0	5.315044	5.465180
24	Rate_Competence	mm	Main environmental concern	Local concern	5.308330	0.03985584	133.1883	0	5.230214	5.386446
25	Rate_Competence	mm	Argumentation style	NoNo	5.339831	0.03901047	136.8820	0	5.263372	5.416290
26	Rate_Competence	mm	Argumentation style	YesBut	5.360444	0.03771155	142.1433	0	5.286530	5.434357

Note. The "estimate" represents the Marginal Mean.

Figure 24

Marginal Mean calculations from R for the profile ratings on environmentalists' Friendliness

	outcome	statistic	feature	level	estimate	std.error	z	p	lower	upper
1	Rate_Friendly	mm	Age	23	5.240464	0.04032917	129.9423	0	5.161421	5.319508
2	Rate_Friendly	mm	Age	42	5.216612	0.04051644	128.7530	0	5.137202	5.296023
3	Rate_Friendly	mm	Age	64	5.142696	0.04151810	123.8664	0	5.061322	5.224070
4	Rate_Friendly	mm	Gender identity	Man	5.193477	0.04017147	129.2827	0	5.114742	5.272212
5	Rate_Friendly	mm	Gender identity	Woman	5.273701	0.03983692	132.3823	0	5.195622	5.351780
6	Rate_Friendly	mm	Gender identity	Non-binary	5.129291	0.04379790	117.1127	0	5.043448	5.215133
7	Rate_Friendly	mm	Race/Ethnicity	White	5.204854	0.04215732	123.4626	0	5.122227	5.287481
8	Rate_Friendly	mm	Race/Ethnicity	Black/African American	5.213752	0.04514934	115.4779	0	5.125261	5.302243
9	Rate_Friendly	mm	Race/Ethnicity	Hispanic/Latino	5.193759	0.04345524	119.5197	0	5.108588	5.278930
10	Rate_Friendly	mm	Race/Ethnicity	Asian	5.188982	0.04470066	116.0829	0	5.101371	5.276594
11	Rate_Friendly	mm	Religiosity	Not religious	5.158951	0.04191907	123.0693	0	5.076792	5.241111
12	Rate_Friendly	mm	Religiosity	Moderately religious	5.244077	0.04003978	130.9717	0	5.165601	5.322554
13	Rate_Friendly	mm	Religiosity	Very religious	5.197687	0.04112841	126.3770	0	5.117077	5.278297
14	Rate_Friendly	mm	Occupation	Office clerk	5.225028	0.04031710	129.5983	0	5.146008	5.304048
15	Rate_Friendly	mm	Occupation	Cleaner	5.224412	0.04218808	123.8362	0	5.141724	5.307099
16	Rate_Friendly	mm	Occupation	Corporate CEO	5.152066	0.04132256	124.6793	0	5.071075	5.233057
17	Rate_Friendly	mm	Political orientation	Liberal	5.201533	0.04039089	128.7799	0	5.122369	5.280698
18	Rate_Friendly	mm	Political orientation	Moderate	5.225485	0.03784386	138.0801	0	5.151312	5.299657
19	Rate_Friendly	mm	Political orientation	Conservative	5.174010	0.04446878	116.3515	0	5.086853	5.261167
20	Rate_Friendly	mm	Type of pro-environmental behavior	Radical behavior	5.134973	0.04284252	119.8569	0	5.051003	5.218942
21	Rate_Friendly	mm	Type of pro-environmental behavior	Moderate behavior	5.175761	0.04075622	126.9932	0	5.095880	5.255642
22	Rate_Friendly	mm	Type of pro-environmental behavior	Private behavior	5.288181	0.04074980	129.7720	0	5.208313	5.368049
23	Rate_Friendly	mm	Main environmental concern	Global concern	5.221581	0.03708137	140.8141	0	5.148902	5.294259
24	Rate_Friendly	mm	Main environmental concern	Local concern	5.178289	0.03761904	137.6507	0	5.104557	5.252021
25	Rate_Friendly	mm	Argumentation style	NoNo	5.168812	0.03817605	135.3941	0	5.093988	5.243636
26	Rate_Friendly	mm	Argumentation style	YesBut	5.232163	0.03643520	143.6019	0	5.160751	5.303574

Note. The "estimate" represents the Marginal Mean.

Figure 25

Marginal Mean calculations from R for the profile ratings on environmentalists' Trustworthiness

	outcome	statistic	feature	level	estimate	std.error	z	p	lower	upper
1	Rate_Trust	mm	Age	23	5.242123	0.04099303	127.8784	0	5.161778	5.322468
2	Rate_Trust	mm	Age	42	5.218241	0.04200192	124.2382	0	5.135919	5.300563
3	Rate_Trust	mm	Age	64	5.188945	0.04342587	119.4897	0	5.103832	5.274058
4	Rate_Trust	mm	Gender identity	Man	5.237700	0.04135709	126.6458	0	5.156642	5.318759
5	Rate_Trust	mm	Gender identity	Woman	5.303160	0.03952998	134.1554	0	5.225683	5.380637
6	Rate_Trust	mm	Gender identity	Non-binary	5.102403	0.04568341	111.6905	0	5.012865	5.191941
7	Rate_Trust	mm	Race/Ethnicity	White	5.214133	0.04463480	116.8176	0	5.126650	5.301615
8	Rate_Trust	mm	Race/Ethnicity	Black/African American	5.231689	0.04565218	114.5989	0	5.142212	5.321166
9	Rate_Trust	mm	Race/Ethnicity	Hispanic/Latino	5.204644	0.04464098	116.5889	0	5.117150	5.292139
10	Rate_Trust	mm	Race/Ethnicity	Asian	5.216526	0.04624842	112.7936	0	5.125881	5.307172
11	Rate_Trust	mm	Religiosity	Not religious	5.208589	0.04336854	120.1006	0	5.123588	5.293590
12	Rate_Trust	mm	Religiosity	Moderately religious	5.225344	0.04176300	125.1190	0	5.143490	5.307198
13	Rate_Trust	mm	Religiosity	Very religious	5.215859	0.04158524	125.4257	0	5.134353	5.297365
14	Rate_Trust	mm	Occupation	Office clerk	5.241863	0.04178583	125.4459	0	5.159964	5.323762
15	Rate_Trust	mm	Occupation	Cleaner	5.248495	0.04329871	121.2160	0	5.163631	5.333359
16	Rate_Trust	mm	Occupation	Corporate CEO	5.159780	0.04401756	117.2209	0	5.073507	5.246052
17	Rate_Trust	mm	Political orientation	Liberal	5.226177	0.04184728	124.8869	0	5.144158	5.308197
18	Rate_Trust	mm	Political orientation	Moderate	5.260388	0.04049251	129.9101	0	5.181024	5.339752
19	Rate_Trust	mm	Political orientation	Conservative	5.162856	0.04451212	115.9876	0	5.075613	5.250098
20	Rate_Trust	mm	Type of pro-environmental behavior	Radical behavior	5.169399	0.04244225	121.7984	0	5.086214	5.252584
21	Rate_Trust	mm	Type of pro-environmental behavior	Moderate behavior	5.186100	0.04302408	120.5395	0	5.101774	5.270426
22	Rate_Trust	mm	Type of pro-environmental behavior	Private behavior	5.291959	0.04127864	128.2009	0	5.211054	5.372864
23	Rate_Trust	mm	Main environmental concern	Global concern	5.246121	0.03870194	135.5519	0	5.170266	5.321975
24	Rate_Trust	mm	Main environmental concern	Local concern	5.185827	0.03840048	135.0459	0	5.110564	5.261091
25	Rate_Trust	mm	Argumentation style	NoNo	5.210004	0.03862586	134.8838	0	5.134298	5.285709
26	Rate_Trust	mm	Argumentation style	YesBut	5.223290	0.03835164	136.1947	0	5.148122	5.298458

Note. The "estimate" represents the Marginal Mean.

Figure 26

Marginal Mean calculations from R for the profile ratings on the profiles' Typicality as environmentalist

	outcome	statistic	feature	level	estimate	std.error	z	p	lower	upper
1	Rate_Typicality	mm	Age	23	5.027087	0.04422672	113.66629	0	4.940404	5.113770
2	Rate_Typicality	mm	Age	42	5.028230	0.04320458	116.38187	0	4.943551	5.112910
3	Rate_Typicality	mm	Age	64	4.963903	0.04329184	114.66141	0	4.879053	5.048753
4	Rate_Typicality	mm	Gender identity	Man	4.999447	0.04404419	113.50981	0	4.913122	5.085772
5	Rate_Typicality	mm	Gender identity	Woman	5.062667	0.04320254	117.18449	0	4.977992	5.147343
6	Rate_Typicality	mm	Gender identity	Non-binary	4.954805	0.04611699	107.43990	0	4.864418	5.045193
7	Rate_Typicality	mm	Race/Ethnicity	White	5.015703	0.04766610	105.22578	0	4.922279	5.109127
8	Rate_Typicality	mm	Race/Ethnicity	Black/African American	4.973094	0.04840508	102.73910	0	4.878222	5.067966
9	Rate_Typicality	mm	Race/Ethnicity	Hispanic/Latino	4.979681	0.04725813	105.37193	0	4.887056	5.072305
10	Rate_Typicality	mm	Race/Ethnicity	Asian	5.060444	0.04692649	107.83769	0	4.968470	5.152418
11	Rate_Typicality	mm	Religiosity	Not religious	5.025098	0.04382034	114.67500	0	4.939211	5.110984
12	Rate_Typicality	mm	Religiosity	Moderately religious	5.022039	0.04408073	113.92821	0	4.935642	5.108435
13	Rate_Typicality	mm	Religiosity	Very religious	4.973568	0.04451201	111.73542	0	4.886326	5.060810
14	Rate_Typicality	mm	Occupation	Office clerk	5.071268	0.04161078	121.87390	0	4.989713	5.152824
15	Rate_Typicality	mm	Occupation	Cleaner	5.056924	0.04386066	115.29521	0	4.970959	5.142889
16	Rate_Typicality	mm	Occupation	Corporate CEO	4.893113	0.04837491	101.14980	0	4.798300	4.987926
17	Rate_Typicality	mm	Political orientation	Liberal	5.087623	0.04281044	118.84072	0	5.003716	5.171530
18	Rate_Typicality	mm	Political orientation	Moderate	5.081440	0.03932769	129.20772	0	5.004360	5.158521
19	Rate_Typicality	mm	Political orientation	Conservative	4.849414	0.05181832	93.58494	0	4.747852	4.950976
20	Rate_Typicality	mm	Type of pro-environmental behavior	Radical behavior	4.969399	0.04534367	109.59410	0	4.880527	5.058271
21	Rate_Typicality	mm	Type of pro-environmental behavior	Moderate behavior	5.013785	0.04408139	113.73928	0	4.927387	5.100183
22	Rate_Typicality	mm	Type of pro-environmental behavior	Private behavior	5.037237	0.04361511	115.49294	0	4.951753	5.122721
23	Rate_Typicality	mm	Main environmental concern	Global concern	5.054854	0.03942262	128.22217	0	4.977587	5.132121
24	Rate_Typicality	mm	Main environmental concern	Local concern	4.956653	0.04015843	123.42745	0	4.877944	5.035362
25	Rate_Typicality	mm	Argumentation style	NoNo	5.028319	0.03839767	130.95377	0	4.953061	5.103577
26	Rate_Typicality	mm	Argumentation style	YesBut	4.985213	0.04164259	119.71428	0	4.903595	5.066831

Note. The "estimate" represents the Marginal Mean.

Figure 27

Marginal Mean calculations from R for the profile ratings on the participants' Identification with the profiles

	outcome	statistic	feature	level	estimate	std.error	z	p	lower	upper
1	Rate_Identification	mm	Age	23	4.631841	0.05345452	86.65013	0	4.527072	4.736610
2	Rate_Identification	mm	Age	42	4.599349	0.05252847	87.55916	0	4.496395	4.702302
3	Rate_Identification	mm	Age	64	4.602369	0.05088231	90.45125	0	4.502641	4.702096
4	Rate_Identification	mm	Gender identity	Man	4.623549	0.05212148	88.70717	0	4.521393	4.725705
5	Rate_Identification	mm	Gender identity	Woman	4.686663	0.05057694	92.66403	0	4.587534	4.785792
6	Rate_Identification	mm	Gender identity	Non-binary	4.517735	0.05539170	81.55977	0	4.409169	4.626300
7	Rate_Identification	mm	Race/Ethnicity	White	4.614561	0.05831106	79.13697	0	4.500273	4.728849
8	Rate_Identification	mm	Race/Ethnicity	Black/African American	4.594170	0.05475271	83.90763	0	4.486857	4.701484
9	Rate_Identification	mm	Race/Ethnicity	Hispanic/Latino	4.560232	0.05600355	81.42756	0	4.450467	4.669997
10	Rate_Identification	mm	Race/Ethnicity	Asian	4.678653	0.05528105	84.63395	0	4.570305	4.787002
11	Rate_Identification	mm	Religiosity	Not religious	4.649191	0.05341976	87.03130	0	4.544490	4.753892
12	Rate_Identification	mm	Religiosity	Moderately religious	4.633609	0.04997384	92.72069	0	4.535662	4.731556
13	Rate_Identification	mm	Religiosity	Very religious	4.551211	0.05461439	83.33356	0	4.444169	4.658254
14	Rate_Identification	mm	Occupation	Office clerk	4.588664	0.05235824	87.63977	0	4.486044	4.691285
15	Rate_Identification	mm	Occupation	Cleaner	4.707718	0.05208496	90.38535	0	4.605633	4.809802
16	Rate_Identification	mm	Occupation	Corporate CEO	4.536088	0.05559054	81.59821	0	4.427133	4.645044
17	Rate_Identification	mm	Political orientation	Liberal	4.675794	0.05239250	89.24549	0	4.573107	4.778481
18	Rate_Identification	mm	Political orientation	Moderate	4.626039	0.04989723	92.71133	0	4.528242	4.723836
19	Rate_Identification	mm	Political orientation	Conservative	4.530396	0.05816987	77.88218	0	4.416385	4.644407
20	Rate_Identification	mm	Type of pro-environmental behavior	Radical behavior	4.451366	0.05495934	80.99381	0	4.343648	4.559084
21	Rate_Identification	mm	Type of pro-environmental behavior	Moderate behavior	4.583573	0.05445143	84.17726	0	4.476850	4.690296
22	Rate_Identification	mm	Type of pro-environmental behavior	Private behavior	4.794927	0.05060533	94.75143	0	4.695743	4.894112
23	Rate_Identification	mm	Main environmental concern	Global concern	4.655359	0.04652664	100.05793	0	4.564169	4.746550
24	Rate_Identification	mm	Main environmental concern	Local concern	4.565021	0.04837903	94.35948	0	4.470200	4.659842
25	Rate_Identification	mm	Argumentation style	NoNo	4.593601	0.04875907	94.21018	0	4.498035	4.689167
26	Rate_Identification	mm	Argumentation style	YesBut	4.628835	0.04709940	98.27801	0	4.536522	4.721149

Further plots of subgroup comparisons

Figure 28

Subgroup comparison across all attributes between participants' different levels of concern

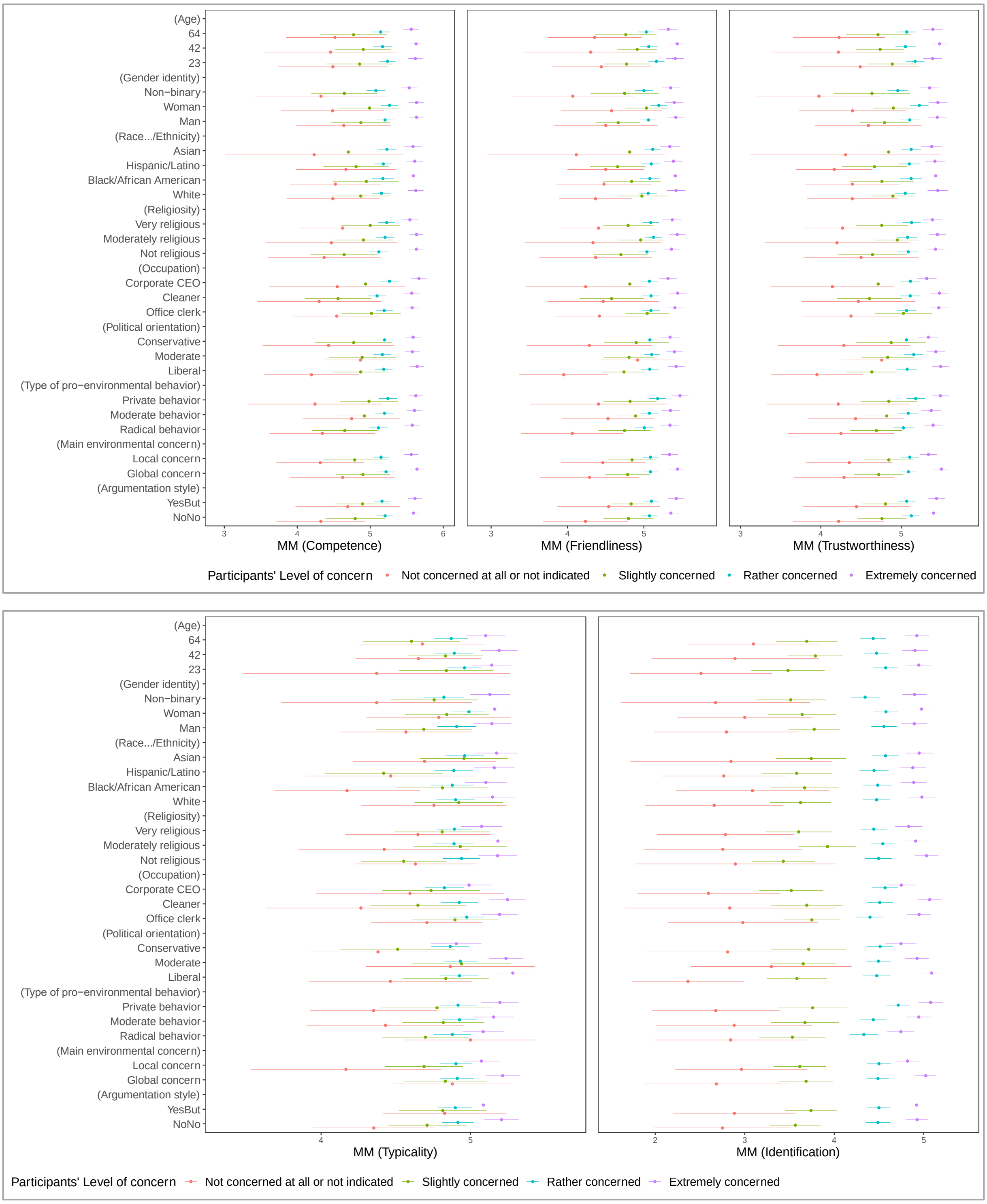

Figure 29

Subgroup comparison matching the profiles' and participants' pro-environmental behaviors

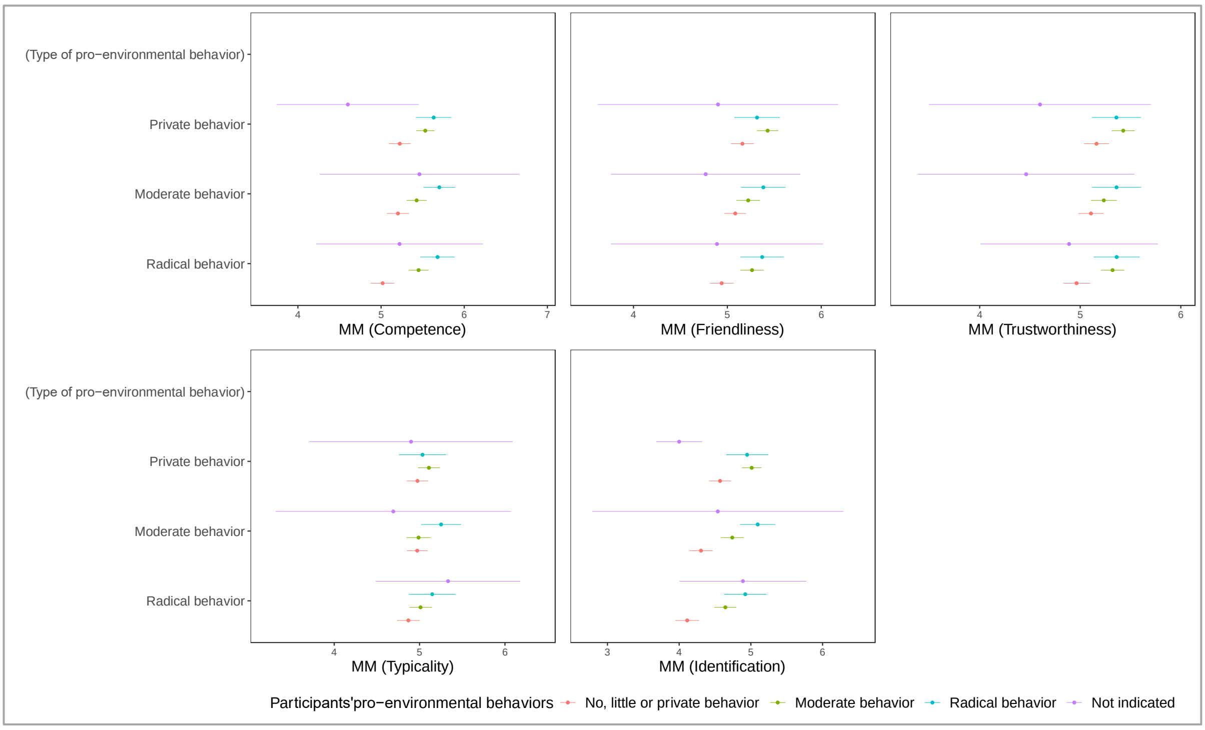

Figure 30

Subgroup comparison matching the profiles' occupation with participants' social class by income

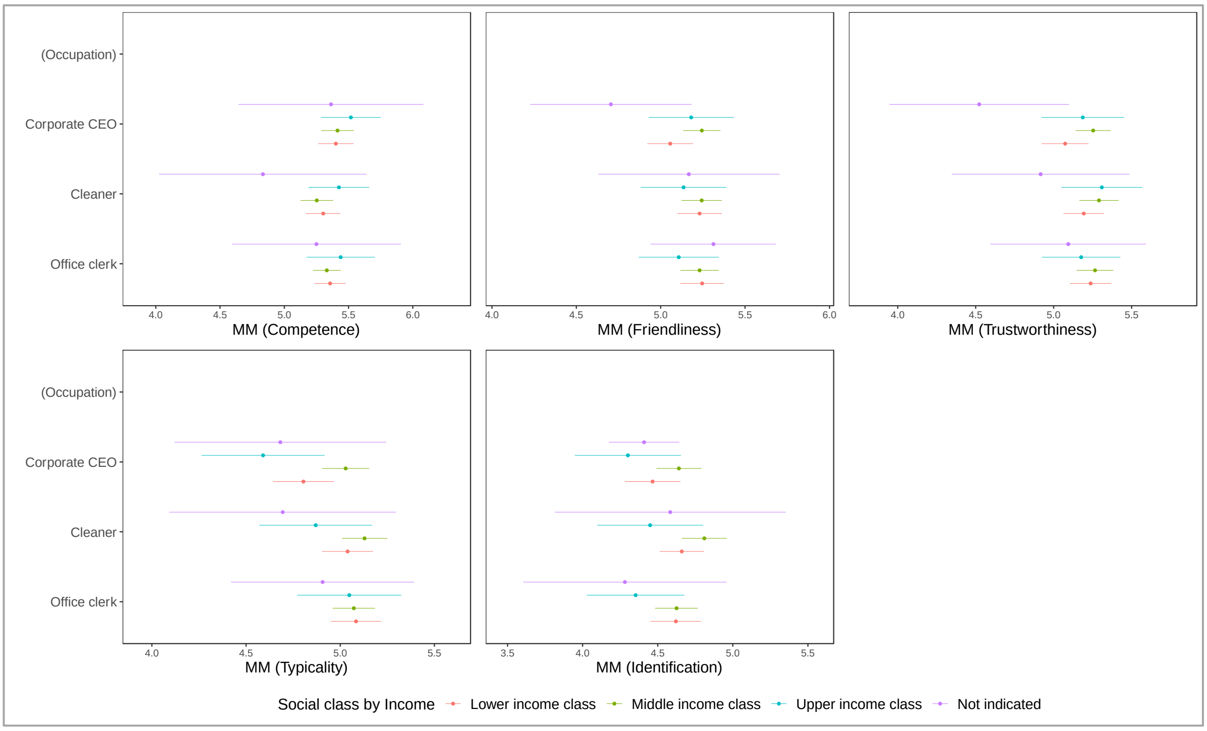

Figure 31

Subgroup comparison across all attributes between participants' different self-assessed social class

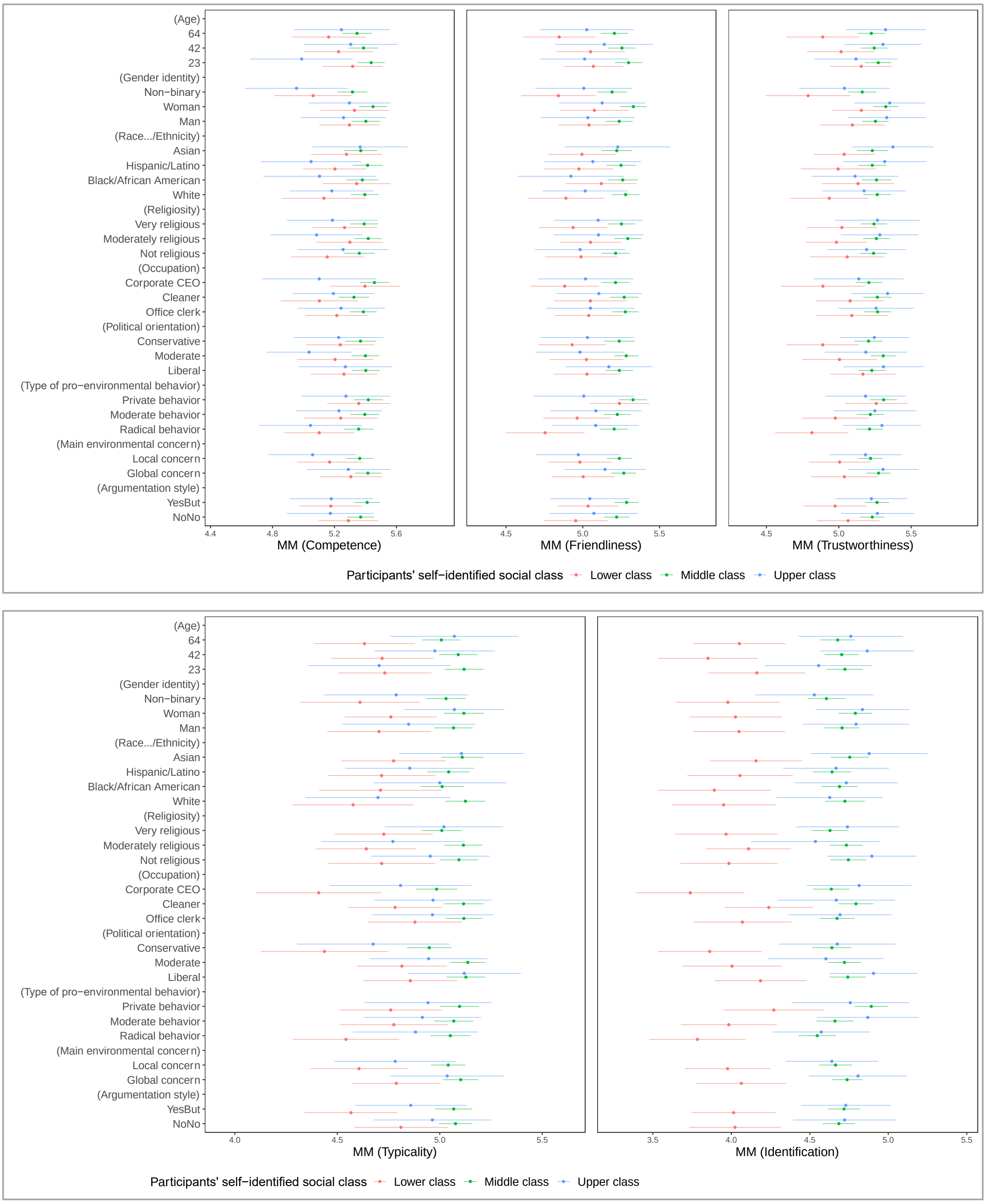

Figure 32

Subgroup comparison across all attributes between participants' different political orientation

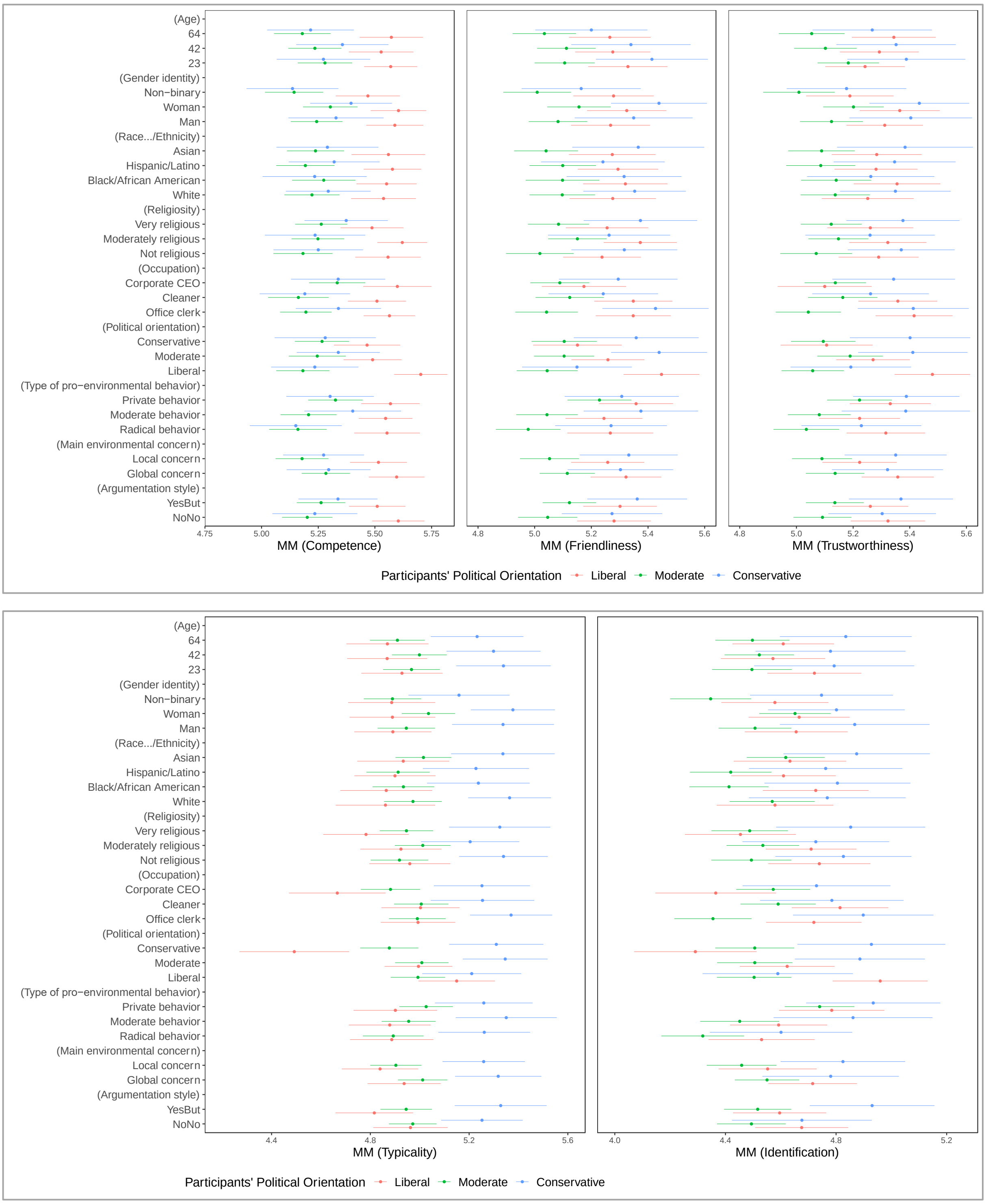

Figure 33

Subgroup comparison across all attributes between participants' memberships in racial-ethnic majority and minority

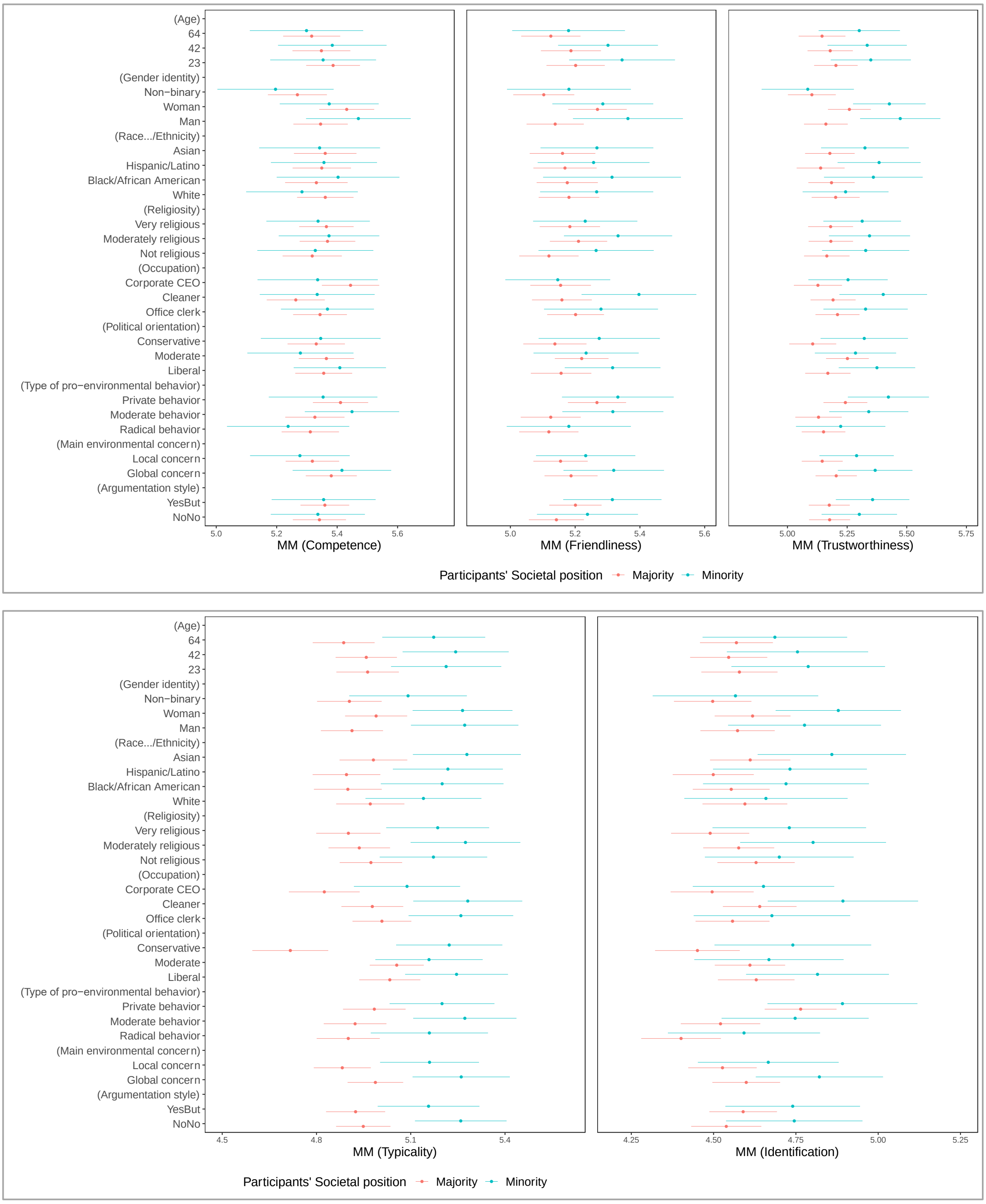

Figure 34

Subgroup comparison matching the profiles' and participants' racial-ethnic group memberships

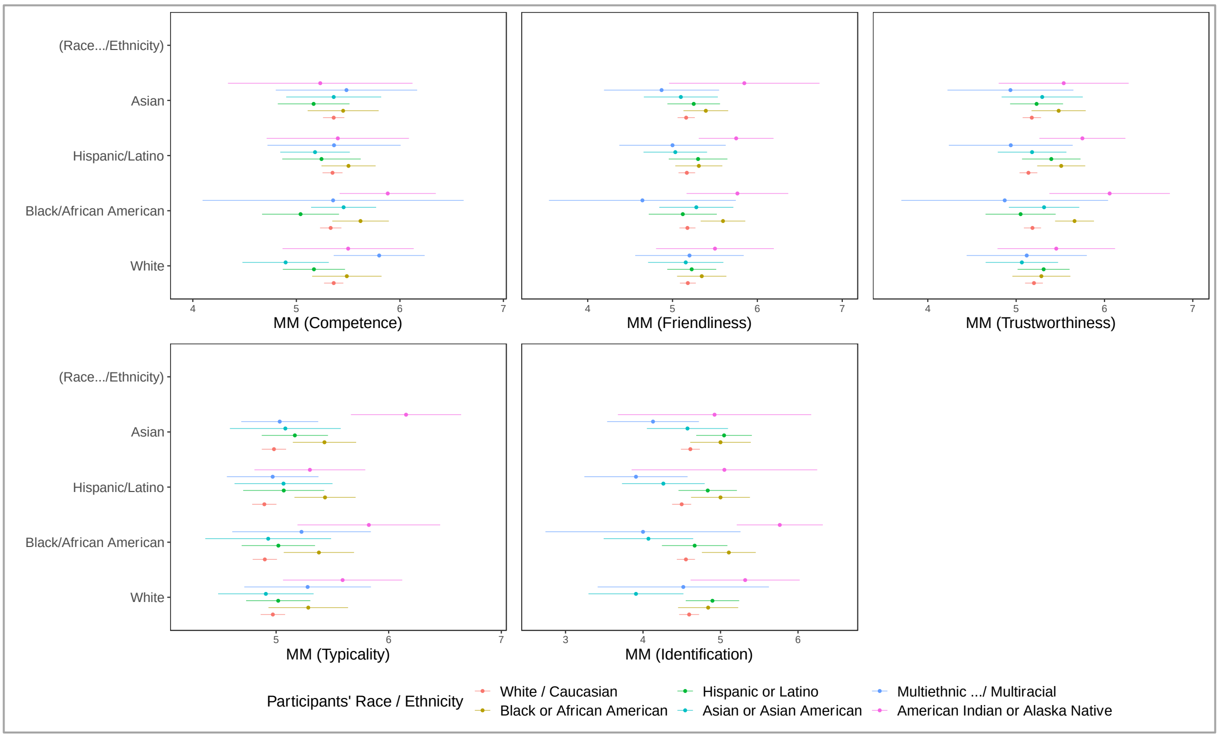

Figure 35

Subgroup comparison matching the profiles' and participants' age

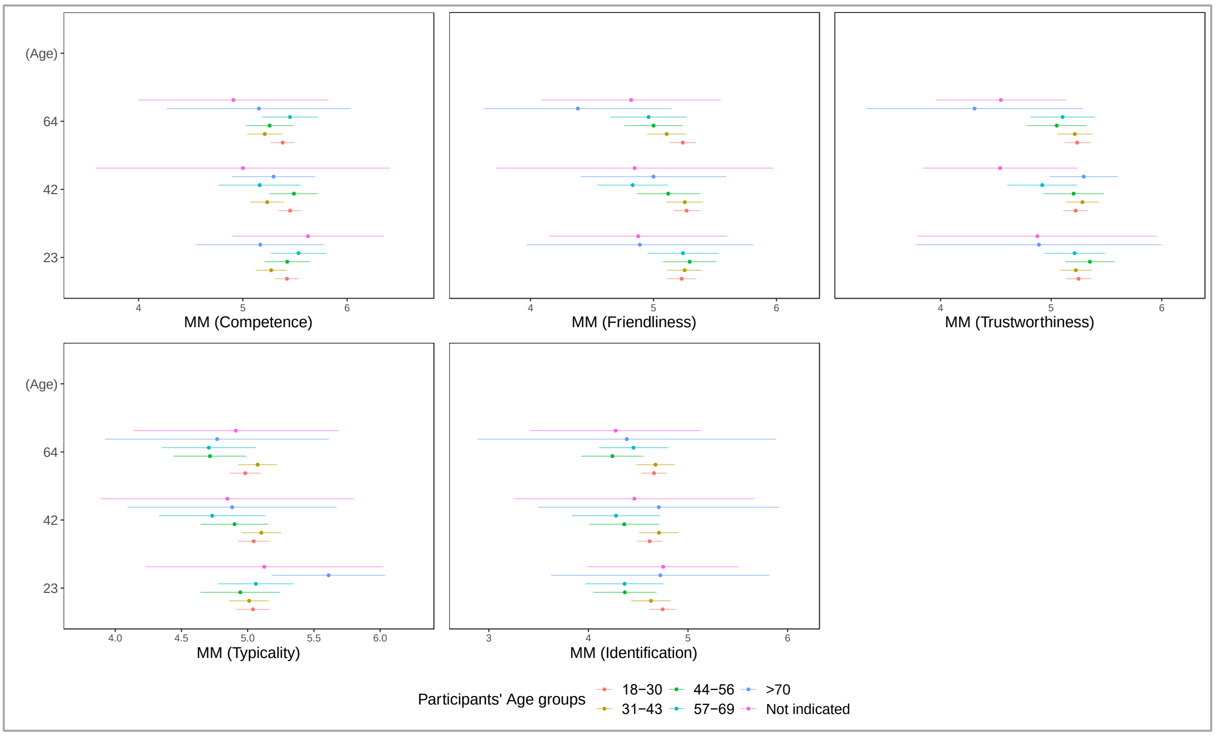

Figure 36

Subgroup comparison matching the profiles' and participants' gender identity

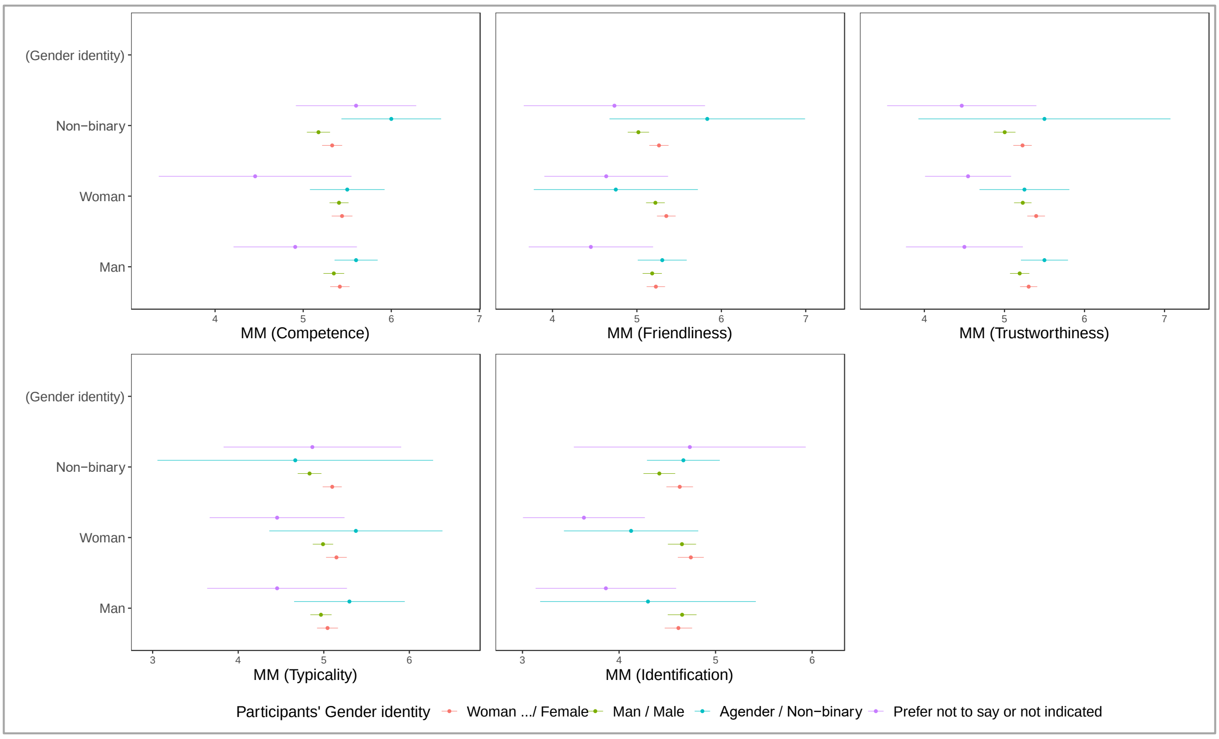

Figure 37

Subgroup comparison matching the profiles' and participants' level of religiosity

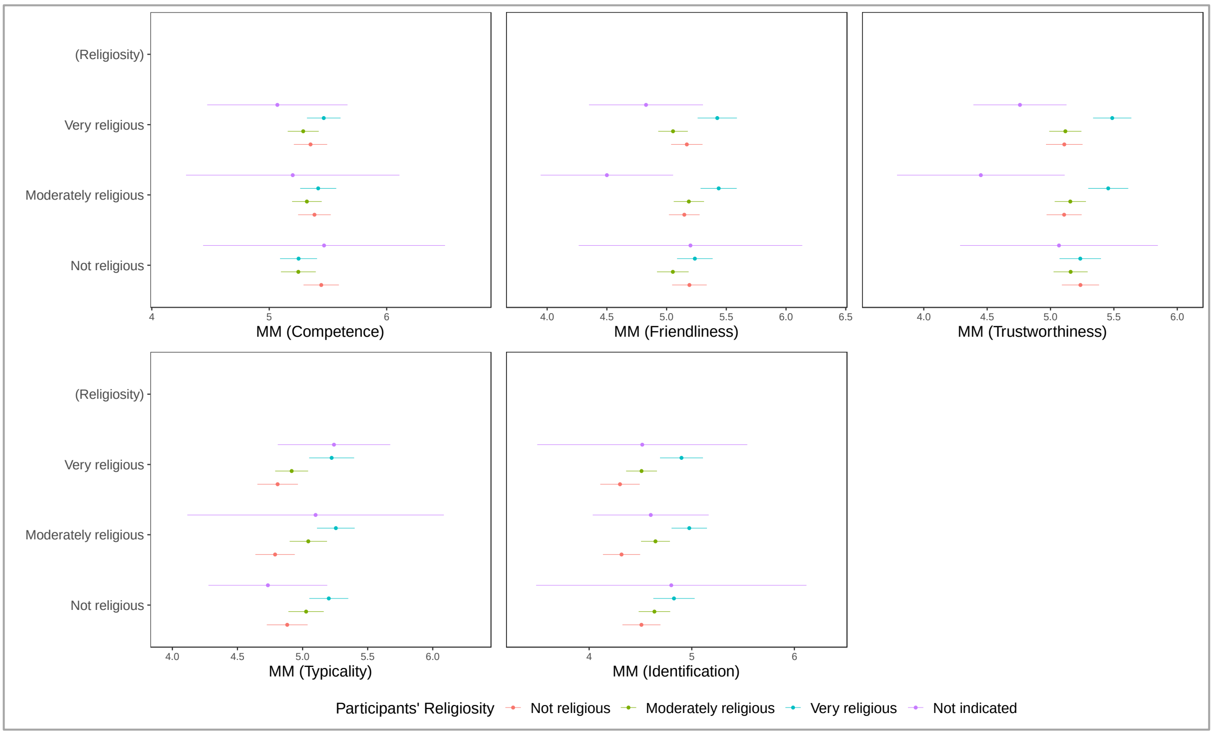

Supplement: SUPPLEMENTARY PRESENTATION 6 — Further results of the conjoint analyses. [file Presentation_6.pdf]
